# Stimulus Distribution Shapes Color and Vibrotactile Perception

**DOI:** 10.1523/ENEURO.0121-25.2025

**Published:** 2025-10-10

**Authors:** Avi Aizenman, Kani Gaff, Dimitris Voudouris

**Affiliations:** Experimental Psychology, Justus Liebig University, Giessen 35394, Germany

**Keywords:** central tendency bias, color perception, psychophysics, tactile perception

## Abstract

Perception is shaped by both the physical properties of stimuli and their contextual presentation, often leading to systematic biases such as the central tendency effect, where perceptual judgments shift toward the average of the stimulus set. This study explored the central tendency bias in vibrotactile perception, an area that has received limited attention while also replicating its well-documented occurrence in color perception to validate previous findings. Using a within-subject design, participants (5 males, 15 females) completed color and vibrotactile discrimination tasks, each consisting of three blocks, which comprised systematically shifted stimulus sets. In an established virtual reality color task, stimuli ranged from yellow–green to blue–green, while in the vibrotactile task, stimuli varied in vibration intensity around a baseline distribution. As predicted, the point of subjective equality shifted toward the mean of the stimulus sets in both tasks, confirming the presence of a central tendency bias. These findings demonstrate that perception of both color and vibrotactile intensity is not determined solely based on the physical properties of the stimulus per se, but it is rather influenced by the distribution of the presented stimuli, underscoring the pervasive role of contextual factors in shaping sensory judgments.

## Significance Statement

Our senses do not work like measuring devices—they integrate incoming information with past experience and surrounding context. What we perceive can be biased toward the average of recently encountered stimuli, a phenomenon called the central tendency bias. Although this bias is well established in vision and hearing, it has never been directly tested for how we perceive touch intensity, despite the critical role of tactile information in daily activities and motor control. By determining whether touch perception is shaped by stimulus context, this research clarifies whether widely used tactile sensitivity measures reflect stable sensory thresholds or are influenced by prior experience. These findings have implications for experimental design, sensory assessment, and technologies that rely on accurate touch feedback.

## Introduction

The accuracy of our own perception is a central and ongoing question. Perception is not only influenced by the physical properties of a stimulus but also by contextual information (Olkkonen and Allred, 2014; Olkkonen et al., 2014) and previous experiences. These influences can span from short-term histories, such as the course of an experiment (Jazayeri and Shadlen, 2010; Chopin and Mamassian, 2012; Olkkonen et al., 2014; Voudouris et al., 2024) to a lifetime (Hansen et al., 2006; Girshick et al., 2011). For example, a stimulus' perceived properties may be biased toward the average of previously encountered stimuli—a phenomenon known as the central tendency bias. This effect was first observed by Hollingworth (Hollingworth, 1910), who showed that participants' size estimates were biased toward the average size of the presented objects. Since then, similar effects have been observed in various perceptual domains, including color (Olkkonen et al., 2014; Aizenman et al., 2024), auditory perception (Marks, 1993), shape and gray value (Huttenlocher et al., 2000), line length (Ashourian and Loewenstein, 2011), interval duration (Jazayeri and Shadlen, 2010), and numerosity (Xiang et al., 2021).

Central tendency biases are typically investigated by assessing how the perception of certain stimulus properties is influenced by the statistical properties of the broader stimulus set. For instance, participants' discrimination thresholds for hue shift toward the mean hue of the variable set (Olkkonen et al., 2014; Aizenman et al., 2024). Similarly, an auditory tone is perceived as louder when embedded in a high-intensity stimulus set, compared with the same tone in a low-intensity context (Marks, 1993). These findings suggest that perception reflects more than a purely physiological process — it is also shaped by prior exposure to stimuli with similar properties. These biases in the perception of stimulus properties can have significant implications when assessing human perception, as using different sets of stimuli to investigate the same question can lead to inconsistent results for an otherwise comparable process.

While central tendency effects are well documented across vision and audition, they have not yet been directly tested in the vibrotactile domain, particularly regarding the perception of tactile intensity. This is important as tactile signals are used as proxies to assess basic processes of perception and action (Colino et al., 2017; Schuetz et al., 2022; Voudouris and Fiehler, 2022), as auxiliary cues during human actions (Mikula et al., 2018), and as feedback in prosthetic design (Moore et al., 2021). If perception of tactile intensity is a contextual measure, it should be influenced by the underlying stimulus distribution. If it represents the detection thresholds per se, it should remain unaffected by contextual stimulus statistics. This is important to distinguish, especially when assessing perceptual thresholds (e.g., detection or discrimination thresholds) because the thresholds themselves may eventually be dependent on the stimulus set.

Previous studies assessing response biases in tactile localization suggest that context influences vibrotactile perception, potentially biasing responses toward the mean of the underlying stimulus distribution (Steenbergen et al., 2014; Brooks et al., 2019). For example, weak tactile stimuli on the forearm tend to be localized toward the center of the tactile stimuli distribution (Brooks et al., 2019). The tactile system is highly adaptable—neural and perceptual processes are shaped by repeated exposure to specific tactile properties (Hollins et al., 1990, 1991; Craig, 1993). The sensitivity to tactile stimulation may depend on the characteristics of the stimuli used to assess such sensitivity. This is important to address, especially considering the continuous effort to characterize tactile sensitivity measures under various contexts (Cai et al., 2023; Bernard et al., 2024; Voudouris et al., 2024).

The present study investigates whether the central tendency biases also influence the perception of vibrotactile intensity. We first examine whether observers demonstrate a central tendency bias in the visual domain, replicating a color perception task modeled after Aizenman et al. (2024). By testing color perception, we can replicate the central tendency bias in a new cohort of observers. We then examine whether these same participants show a comparable bias in vibrotactile intensity perception. If participants' perceptual thresholds shift with the stimulus distribution in both tasks, this would suggest that central tendency biases generalize across modalities. Conversely, if the effect is present in color but absent in the vibrotactile task, this points to the possibility of modality-specific processing in perceptual decision-making. In this case, robust vibrotactile thresholds across ranges of different stimuli may indicate that these thresholds are based on the actual signal that activates the receptors without any major influences of the underlying context.

## Materials and Methods

### Participants

The study was conducted at the host university, using a within-subject design with two tasks in two separate experimental sessions: a color and a tactile task. Twenty-one volunteers participated in the study. Three participants were excluded from the analysis due to the exclusion criteria mentioned below. One observer was not able to complete the color session. This resulted in a total sample of 19 participants for the color task, and a sample of 20 participants for the tactile task (5 males, 15 females), with a mean age of 25.05 years (range, 20–35 years). Participants were naive to the study's purpose prior to participation. After providing informed consent, participants completed the Edinburgh Handedness Inventory (Oldfield, 1971), which revealed that 18 were right-handed. The study protocol was approved by the local ethics committee and conducted in accordance with the Declaration of Helsinki (2013, except for §35, preregistration). Participants were compensated with either course credits or €8 per hour. Participants completed both experimental tasks. The order was counterbalanced so that 10 participants began with the color experimental task, and the remaining 9 started with the vibrotactile task.

### Color task

#### Apparatus

This study used the HTC Vive Pro Eye headset, which features two OLED screens (with a resolution of 1,440 × 1,600 pixels per eye) to present a color-swiping game. The experiment ran on a PC with Windows 10 64 bit, powered by an AMD Ryzen Threadripper 3,990× 64-core processor (2.9 GHz), 256 GB RAM, and an NVIDIA GeForce RTX 3090 graphics card. The color task was developed using Unity (version 2019.3.8f1). The game maintained a frame rate of ∼90 Hz.

### Stimuli

**Figure 1.**
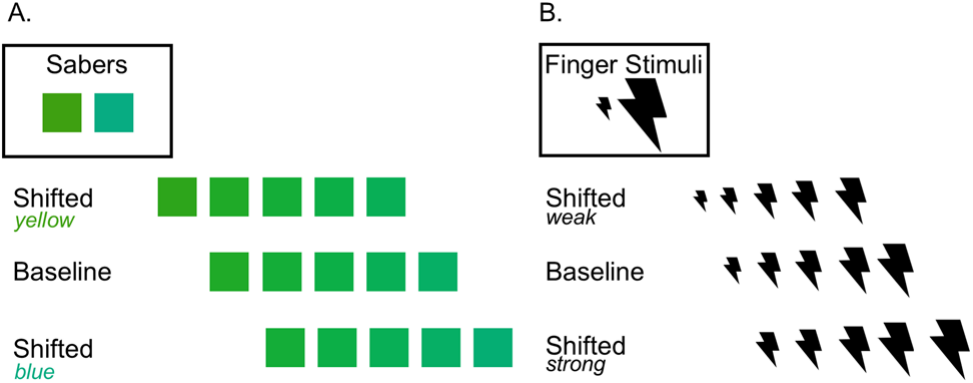
Stimuli for the color and vibrotactile discrimination task. ***A***, The three sets of hues tested and the saber hues for the color discrimination task. The saber hues were consistent across all the blocks of the color task. Each block tested one of the three sets of hues; the hue stimulus set shifted toward yellow, the baseline set of hues, or the hue stimulus set shifted toward blue. Note that the middle hue of each stimulus set was present in all stimulus sets. ***B***, A visual representation of the three vibrotactile stimulus sets. The size of the lightning bolt symbol scales with the intensity of the stimulus. The top row schematizes the set of vibrotactile stimuli shifted toward a weaker intensity, the middle row shows the baseline vibrotactile stimuli set, and the bottom row represents the vibrotactile set shifted toward a stronger intensity.

Nine hues were selected from the green color spectrum, spanning from yellow–green to blue–green. Green was chosen as the test color category as it covers a wide range within the DKL color space and does not overlap with any other color category borders, allowing us to test a sufficient number of distinct hues. For the hue discrimination task, the two most extreme blue–green and yellow–green hues were always used as the standard color stimuli and were presented as the color of two virtual sabers that appeared expanding from the hand-held controllers, with the seven intermediate hues used as test stimuli across three different blocks of trials. We presented three separate blocks of trials, each of which contained five different hues. We selected hues such that the differences between adjacent test colors varied smoothly in fixed multiples of discrimination threshold. The intensity differences between stimuli were set at 1.2 JNDs, based on discrimination data from previous work (Witzel and Gegenfurtner, 2013). Figure 1*A* displays the three sets of stimuli generated from this procedure.

#### Procedure

**Figure 2.**
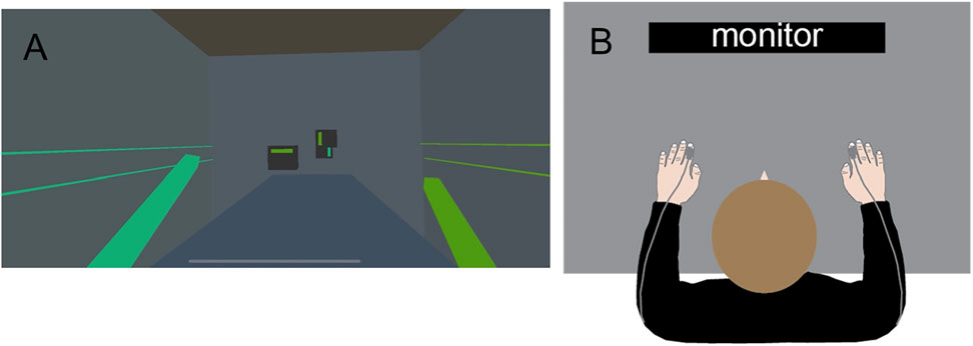
***A***, A screenshot of the color discrimination task. Cubes with a colored stripe gradually approached from the distance, and participants were instructed to swipe the cube perpendicular to the colored stripe with the best matching saber. The closest cube in the above schematic should be swiped in the top–down direction. ***B***, A top-view depiction of the setup used for the tactile task. Participants held their hands with palms facing down on the table. Two tactile actuators (gray boxes) were gently fixed at their two index fingers, and a third one was fixed to their sternum (not shown here).

Participants were instructed through a prerecorded video, which included a brief video clip of the experiment. The participants were instructed to swipe approaching cubes along a plane perpendicular to the colored stripe at their center, using the saber that most closely matched the hue of the stripe. An example of this is shown in Figure 2*A*. In addition to counterbalancing the saber color that would appear in each hand at the beginning of the session, participants were instructed that halfway through the session, the saber color in each hand would swap and that a message would appear in text alerting them to this change. We did these to eliminate any potential response biases due to handedness. Following the instructions, participants were tested for color deficits using the Ishihara color test (Ishihara, 1997). After passing the test, participants placed the headset on, adjusting for comfort.

The experiment consisted of three blocks, each containing a unique set of hues. The order of blocks was counterbalanced using a Latin square design. During the experiment, participants held an HTC Vive controller in each hand, each of which comprised a colored saber of a different shade of green (standard stimuli, yellow–green and blue–green, as shown in Fig. 1*A*). The color of the sabers was consistent across all three blocks. During the experiment, cubes with a colored horizontal or vertical stripe of different shades (comparison stimuli) approached the participant. Participants were instructed to swipe the nearest cube by moving the saber orthogonally to the colored stripe inside the cube. Participants had to use the saber whose color best matched the colored stripe on the cube. This fits typical procedures in sensory discrimination tasks where a “comparison” stimulus of properties that vary from trial to trial is discriminated from a “standard” stimulus of fixed properties (e.g., 27,30). The first minute of each block presented cubes with the endpoint hues (closest to yellow or blue) from the set of colors tested in that block to familiarize participants with the task. After this, the five possible hue stimuli were presented with equal prevalence, with cubes appearing ∼11 m away, and traveling at ∼2 m/s toward the participant. Swipes resulted in visual feedback (fireworks) and an auditory cue if the swipe direction was correct. The cube would then disappear. If the swipe direction was incorrect, the cube disappeared, but no auditory cue was provided. If the saber and cube did not make contact, the cube continued moving past the observer. These “miss” trials, where there was no contact between the saber and cube, were excluded from subsequent analyses. On average, ∼5% of the cubes were missed. All trials where an observer swiped a cube (even if the swipe was in the wrong direction) were included for analysis. Each block lasted 16 min, with ∼55 swipes per minute, totaling 840 trials per block. Participants took a 5 min break between blocks. The color session thus lasted ∼60 min in total.

### Vibrotactile task

#### Apparatus

A custom-made vibrotactile stimulation device (Engineering, 2014) was used to induce vibrotactile stimulation (250 Hz) that lasted for 750 ms.

#### Stimuli

Participants were presented with a vibrotactile stimulus at the dorsal part of the first phalanx of their index finger of each of their hands and a third stimulus at their sternum (∼2 cm below the clavicle). Their task was to tell which of the two stimuli on their fingers was most similar to the stimulus on their sternum. A schematic of the experimental setup is shown in Figure 2*B*.

In total, we presented nine vibrotactile stimuli that varied in intensity (i.e., peak-to-peak displacement of the actuator), ranging from 34 to 96 arbitrary units (one unit corresponds to 3.16 µm of peak-to-peak displacement). Similarly to the color task, two of those stimuli were standard stimuli (weak and strong), applied to the participant's index fingers, while the remaining seven intermediate comparison stimuli were presented on the sternum across three different blocks of trials. As in the color task, we presented three blocks of trials, with each block contained five different vibrotactile intensities. Similar to the color task, the intensity differences between tactile stimuli were set at 1.2 JND, based on data from Voudouris and Fiehler (2017) that employed a similar vibrotactile discrimination task of stimuli with shorter durations (50 ms). A visual representation of the three sets of stimuli is schematized in Figure 1*B*. The stimuli ranged from a strong vibrotactile buzz to a weak yet still noticeable vibrotactile stimulation.

#### Procedure

Similar to the color task, instructions were prerecorded and shown to participants before data collection began. Participants were asked to keep their hands still and identify which hand stimulus best matched the vibrotactile stimulus on the sternum. Observers sat at a desk (117 × 80 cm), with their hands resting palm-down on its surface. Two foot pedals, one for each foot, were placed under the desk. Three vibrotactile stimulators were attached to the participant using hypoallergenic adhesive tape: one on the dorsal side of the proximal phalanx of the right index finger, one on the left index finger, and one on the sternum. Stimuli of fixed intensities were presented on the fingers, similar to the procedure used in the color task. Specifically, one finger received the weakest stimulus, while the other received the strongest. Similarly to the color task, the order of stimulus presentation was counterbalanced across participants, and this order was flipped halfway through each block of trials: Ten participants began with the strongest stimulus on the right finger and the weakest on the left, while the remaining nine participants began with the strongest on the left and the weakest on the right. In each trial, the participant was exposed to vibrotactile stimuli at all three locations simultaneously. The first trial of each block began when the experimenter pressed a button on the keyboard. Approximately 500 ms later, the three stimuli were presented simultaneously for 750 ms. The participant was then asked to respond whether the stimulus on the sternum best matched the vibrotactile stimulus on the right or left hand by pressing the corresponding foot pedal. Participants could respond at their own pace. The next trial began automatically 500 ms after the participant's response.

In the first 40 trials of each block, only the weakest and strongest intensities of that block's stimulus set were presented on the sternum. This served as a familiarization process for the participants, similar to the initial trials in the color task. These 40 familiarization trials took ∼1 min to complete. After this familiarization period, the experiment continued with the sternum receiving one of the five possible variable intensities presented in that block. Each intensity was presented 80 times, resulting in 400 trials. Including the familiarization trials, each block comprised 440 trials, taking ∼12 min to complete. Participants had a 5 min break between blocks.

Each block tested a specific stimulus set, and the block order was counterbalanced across participants using a Latin square design. A practice block preceded the tactile session to ensure participants understood the task. In this block, participants were exposed to 56 trials that included eight random repetitions of the seven possible stimulus intensities that were presented during the actual experiment. The 11 participants who failed to correctly respond in at least 65% of the practice trials repeated the practice block until they reached the required threshold. The tactile session lasted ∼60 min in total.

### Code accessibility

The data files from the color and vibrotactile tasks as well as the output of the data analysis including model parameters can be accessed at https://osf.io/t3f9s/?view_only=c4292bf70b384d369cb291f5e6d93ee5.

Data analysis scripts are available upon request.

## Results

### Analysis

**Figure 3.**
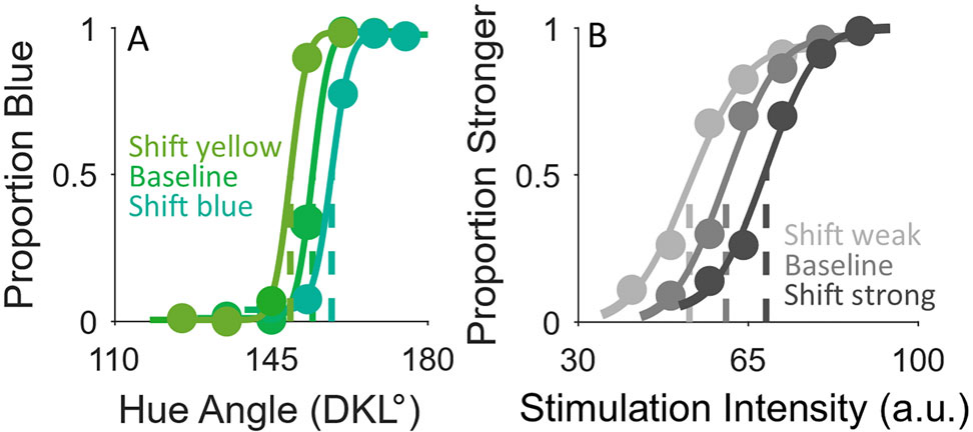
Example psychometric functions for one representative participant for the (***A***) color and (***B***) vibrotactile tasks. Each psychometric function represents one stimulus range and one block of data. The dotted vertical line represents the PSE. In this example, a clear shift of the PSEs can be seen for each task. Colors shown are illustrative and may not match the stimulus hues shown in the experiment.

To analyze participant responses, we fit the discrimination responses of each participant separately for each stimulus range and task with psychometric functions using the psignifit 4 toolbox (Schütt et al., 2016) in MATLAB 2022B (“The MathWorks Inc. (2022). MATLAB version 9.13.0 (R2022b), The MathWorks Inc. https://www.mathworks.com,” n.d.). Psychometric fits were plotted for each participant and stimulus range and task, with representative examples shown in Figure 3, *A* and *B*. Specifically, we fit the responses with a cumulative Gaussian distribution and estimated the decision boundary as the stimulus intensity at 50% correct (i.e., the point of subjective equality; PSE). By fitting the responses separately for each stimulus range and participant, we assessed whether the PSEs shifted with the presented stimulus range. For the data included in the analysis, we applied the following exclusion criteria: PSE values were required to fall within the range of tested stimuli to ensure reliable estimation. Observers with PSE values outside this range were excluded. As a result of these criteria, data from three observers were excluded due to insufficient performance in the practice session of the vibrotactile task.

In addition to calculating the PSEs that are the relevant variables to test our hypotheses, we further explored how discrimination difficulty changed across stimulus ranges and tasks. To this end, we first determined the sigma (*σ*), which reflects the width of the fitted Gaussian. Specifically, this was calculated for each psychometric function as the difference in stimulus intensity between the 50 and 84% points. In addition, we determined the guess and lapse rates, which represent the lower and upper asymptote of the psychometric fit. We do not have a priori hypotheses regarding differences in *σ*, guess, or lapse rate, either between ranges or between the two tasks, but these parameters may shed light on differences in task difficulty between these two modalities.

To examine the effect of stimulus range on sensory perception, as indicated by (color and vibrotactile) discrimination thresholds, we conducted two separate one-way repeated–measures ANOVAs for each task. If the assumption of sphericity was violated, a Greenhouse–Geisser correction was applied if *ε* < 0.75; otherwise, a Huynh–Feldt correction was used. Corrected degrees of freedom were reported. Significant main effects were followed by post hoc tests to compare PSEs across the three stimulus ranges of that task. To account for multiple comparisons, Holm-adjusted *p* values were reported. Effect sizes were provided as *η*^2^ values for the ANOVAs. The alpha level was set at 0.05. Statistical analyses were conducted using JASP Team (2025).

The ANOVA results indicated that the stimulus range affects discrimination thresholds (see Results for details). To explore whether sensory perception shifts fully align with shifts in the presented stimuli, we developed a difference metric to quantify the magnitude of this alignment. This metric calculates the difference between the PSEs in the two shifted conditions, divided by the difference between the central stimulus intensities of the respective distributions. We computed this metric separately for each participant in both the color and vibrotactile datasets. If PSEs remain stable and invariant between the shifted conditions, the difference between PSEs would be 0.0, resulting in a difference metric of 0.0. Conversely, a perfect alignment of PSE shifts with stimulus shifts would yield a difference metric of 1.0, indicating a full central tendency bias where the PSE matches the central tendency of the underlying stimulus distribution. To determine whether the stimulus set induces a central tendency bias, we analyzed the difference metric from each task using two one-sided *t* tests: one against zero (indicating no shift) and one against one (indicating a full shift). This results in two *t* tests per experimental task. This method aligns with equivalence testing using two one-sided tests to assess whether a value falls within a defined range (Lakens et al., 2018). These *t* tests allow us to evaluate whether there is a shift in discrimination thresholds (indicated by a difference metric greater than 0) or whether the shift is incomplete, as previously observed (Aizenman et al., 2024), which would be indicated by a difference metric larger than 0 and smaller than 1.

### PSE

The PSE represents the decision boundary for each set of stimuli tested. In the color task, this is the (comparison) hue on each cube that observers are equally likely to match with the (standard) hue presented on either saber. In the vibrotactile task, this is the stimulus intensity at the sternum which participants are equally likely to match with the “weak” and “strong” standard stimuli on their hands.

#### Color task

**Figure 4.**
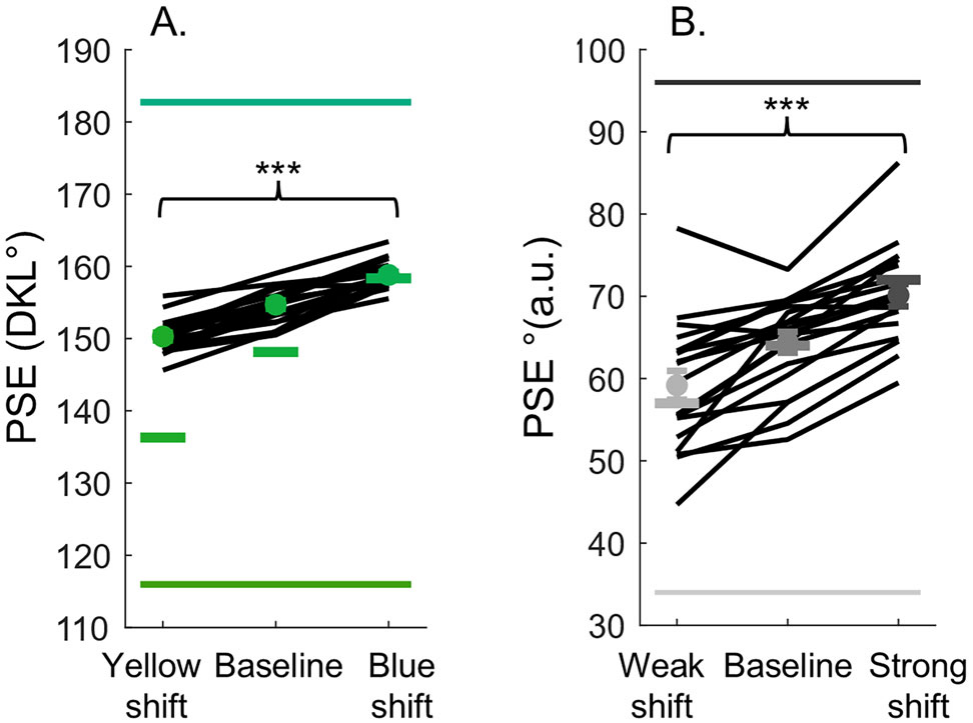
Average and individual PSEs for the color (***A***) and vibrotactile (***B***) tasks. The filled circles represent the mean PSE, and each black line represents the PSE values of a single participant. The thick horizontal bars near the circular markers show the center hue/stimulus intensity of each stimulus set. Error bars denote the SEM; however, these are quite small and may not be visible for most points. The horizontal bars at the bottom and top represent the values of the standard stimuli for each task. In the color task, these are the hues of the sabers used as reference points for comparison, while in the vibrotactile task, they represent the fixed intensity levels of the vibrotactile buzz presented to each finger. If a *p* value is <0.001, it is flagged with three stars (***).

Figure 4*A* shows individual and average PSE values for each stimulus range in the color task. The filled circles represent the mean PSE, while each black line represents one participant. Horizontal bars near the average data points indicate the center hue of each stimulus range. A noticeable shift of the mean PSE in the direction of the shifted stimulus set is observed. This was confirmed by a significant main effect of stimulus range on PSEs (*F*_(2,36)_ = 138.09; *p* < 0.001; $\eta_{p}^{2}=0.89$). Further post hoc tests indicate significant differences between the PSEs of all stimulus ranges, both *t* > 8.01 and both *p* < 0.001.

#### Vibrotactile task

Figure 4*B* shows individual and average PSE values for each stimulus range tested in the vibrotactile task. Similar to the color task, a clear shift of the PSEs in the direction of the shifted stimulus can be observed. This was confirmed by a main effect of stimulus range on PSEs (*F*_(1.60,30.35)_ = 60.56; *p* < 0.001; $\eta_{p}^{2}=0.76$), and post hoc tests revealed significant differences between all stimulus ranges, both *t* > 4.9 and both *p* < 0.001.

### Discrimination performance

**Figure 5.**
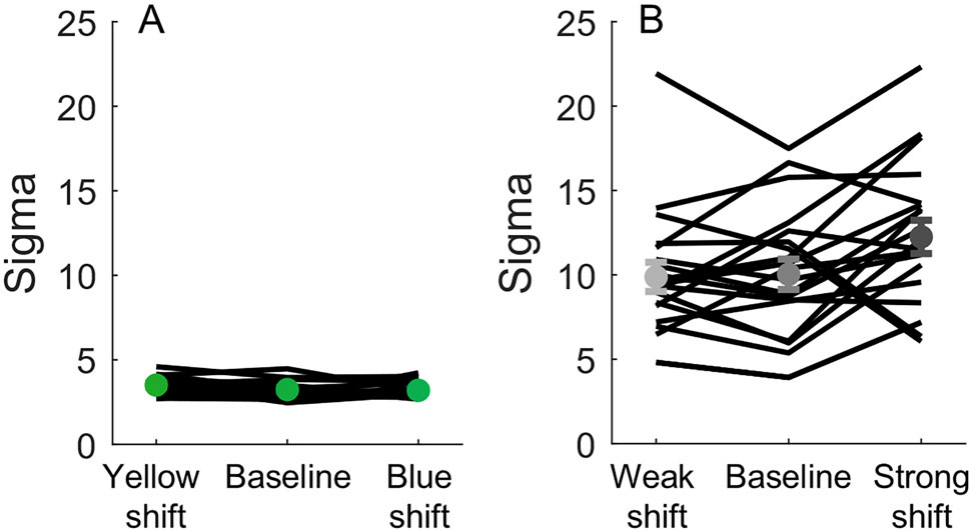
Discrimination variability as reflected in sigma (*σ*) for the color (***A***) and vibrotactile (***B***) tasks. Each black line represents one participant. The green (***A***) and gray (***B***) circular markers represent the mean for that condition, and error bars indicate the SEM. Extended Data Figure 5-1 shows the psychometric model parameters for guess and lapse rates as well.

The psychometric fits also provide a parameter, sigma (*σ*), which reflects discrimination performance. Larger values of *σ* indicate poorer discrimination. Figure 5, *A* and *B*, displays the average *σ* for the color and vibrotactile tasks, respectively. Clear differences emerge between these tasks: the vibrotactile task yields larger and more variable *σ* values, suggesting worse and more variable discrimination performance compared with the color discrimination task. Additional insights into task difficulty are provided by the guess and lapse rates. We observe generally higher and more variable guess rates in the vibrotactile compared with the color task (Extended Data Fig. 5-1*A*,*B*). Lapse rates followed a similar pattern, being larger and more variable in the vibrotactile than the color task (Extended Data Fig. 5-1*C*,*D*). Together, these indicate that the vibrotactile task is associated with poorer and more variable discrimination performance. An important question is whether these differences in task difficulty influence the magnitude of the shift in the PSE.

10.1523/ENEURO.0121-25.2025.f5-1 Figure 5-1 Guess and lapse rates for the color and vibrotactile tasks. Panels A and B show the guess rates for the color and vibrotactile tasks, respectively, while panels C and D show the lapse rates for the color and vibrotactile tasks. Download Figure 5-1, ZIP file.

**Figure 6.**
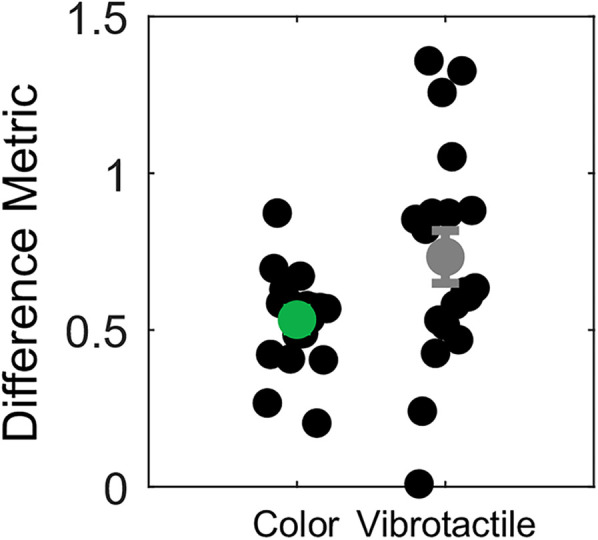
Difference metric for the color and vibrotactile tasks. Each black circle represents one participant for each task. The green and gray circles represent the average difference metric of the color and vibrotactile experiment, respectively. The error bars indicate the SEM.

As described previously, we developed a difference metric to quantify the magnitude of the PSE shift relative to the shift in the tested stimuli. A complete PSE shift, indicating a perfect central tendency bias, corresponds to a metric of 1.0, while a stable PSE (indicating no shift) corresponds to a metric of 0.0. Figure 6 presents the results of this analysis. One-sample, one-sided *t* tests were conducted separately for the color and vibrotactile task to determine if the PSEs differed from 0. The results showed that the PSEs were significantly larger than 0, indicating that the PSEs were not invariant across the three stimulus sets for both color (*t*_(18)_ = 15.34; *p* < 0.001) and vibrotactile stimuli (*t*_(19)_ = 8.97; *p* < 0.001). Additional one-sample, one-sided *t* tests assessed whether the PSEs were smaller than 1. The results revealed that the PSEs were significantly smaller than 1 for both color (*t*_(18)_ = −13.43; *p* < 0.001) and vibrotactile stimuli (*t*_(19)_ = −3.37; *p* = 0.003), indicating that the shift in the PSEs fell short of a full range effect.

In the color task, the difference metric (Fig. 6, green) falls between 0 and 1, indicating only a partial shift in the PSE. This suggests that while the PSEs did not remain stable across the stimulus ranges, they did not fully shift to the center hue of the new stimulus set. Participants adapted their PSEs only partially to the range of the stimulus distribution.

**Figure 7.**
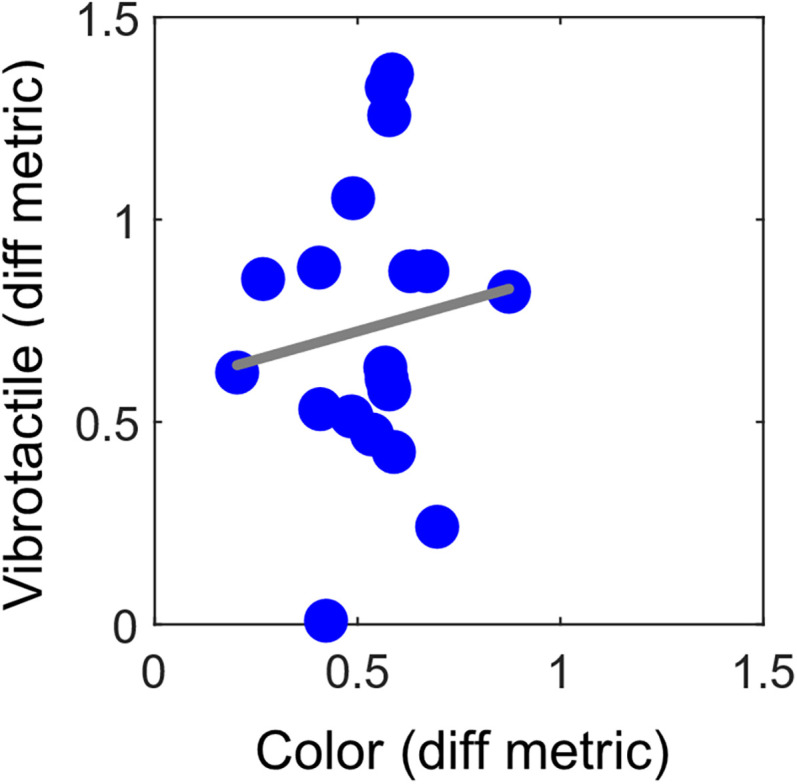
The relationship between the magnitude of the difference metric across the color and vibrotactile tasks. Each blue circle represents the difference metrics for one participant. A value of 1 on the *x*- and *y*-axes reflects a full shift of the PSEs across the stimulus range for both tasks, whereas a value of 0 reflects no shift in the PSEs. The gray line shows the best fit line for a linear regression.

In the vibrotactile task, there was greater variability in the difference metric across participants; some exhibited a full shift in their PSEs, while others showed no shift at all. However, the average difference metric across participants in the vibrotactile task indicated a partial shift in the PSEs. These findings suggest that, on average, participants do not maintain a stable PSE, nor do they fully adapt to the range of stimuli tested. The difference metric results demonstrate substantial variability across participants for the vibrotactile discrimination task. To investigate whether the sensitivity to the stimulus set is an inherent and individual characteristic, we conducted a linear regression across the difference metric values obtained for the color and vibrotactile tasks. The linear regression is presented in Figure 7. The regression equation was not significant (*F*_(1,17)_ = 0.25; *p* = 0.65), with an *R*^2^ = 0.01 and a slope of *b* = 0.28 (SEM, 0.56). We have no evidence of a linear relationship between the difference metric values for the color and vibrotactile tasks.

## Discussion

This study aimed to determine whether sensory perception is influenced by the context in which a stimulus is presented, focusing on how the underlying distribution of stimuli affects color and vibrotactile perception. We conducted a within-subject design experiment with two tasks: color and vibrotactile discrimination. Each task consisted of three blocks, with stimulus sets that were systematically shifted. In the color task, stimuli were shifted toward yellow–green or blue–green hues, while in the vibrotactile task they were shifted toward stronger or weaker vibrations, always relative to a baseline distribution. We predicted that the PSEs would shift toward the center of the presented stimuli sets in the color task, following previous findings (Aizenman et al., 2024), indicating a central tendency bias. If such a bias also arises in the perception of vibrotactile intensity, we additionally expected to observe a shift of the PSEs in the tactile task. Our results confirmed this bias in both tasks showing that the PSEs are not fixed but are shifted toward the center of the underlying stimulus distribution. These highlight that perception of vibrotactile stimuli is not simply the outcome of processing the true sensory signal that arises at the mechanoreceptors but results as the synthesis of that signal with priors about the underlying context.

The impact of the underlying stimulus distribution on vibrotactile perception has not been widely studied. Previous work (Brooks et al., 2019) has noted a potential central tendency bias in the tactile localization of weak stimuli presented on the forearm, but did not delve deeply into these findings. Meanwhile, to the best of our knowledge, there has been no attempt to characterize how the properties of the underlying stimulus distribution influence the perception of vibrotactile intensities. This is a significant gap, as vibrotactile perception plays a critical role in understanding sensory processing (Juravle et al., 2010; Voudouris and Fiehler, 2017) or enhancing human–machine interfaces (Vendrame et al., 2024; Zikmund et al., 2024). Our results demonstrate that vibrotactile perception of intensity is sensitive to the underlying stimulus distribution, analogous to the central tendency bias consistently observed across other sensory systems (Marks, 1993; Jazayeri and Shadlen, 2010; Olkkonen et al., 2014). This demonstrates that estimations of vibrotactile perception are sensitive to the details of the employed experimental paradigms and do not necessarily reflect solely the underlying physiological process, such as the encoding of the stimulus at the receptor level, but are rather influenced by (at least) short-term prior information.

In the color task, our findings align with previous studies (Olkkonen et al., 2014; Aizenman et al., 2024). When examining the extent of the shift, we found that the average PSE shifted only partially, falling approximately between a full shift and stable PSEs. This partial shift is consistent with previous work (Aizenman et al., 2024), which also reported a similar central tendency bias in color perception with a comparable VR setting. Olkkonen and Allred (Olkkonen et al., 2014) additionally demonstrated a central tendency bias in a delayed hue estimation task. However, this bias toward a central value has not always been consistently observed. Olkkonen and Allred (Olkkonen et al., 2014) suggest that previous studies, such as Nilsson and Nelson (Nilsson and Nelson, 1981), often used a wide range of hues, resulting in an average hue close to neutral, which may obscure the bias. Importantly, this explanation does not apply to our study, where each stimulus set had a clearly defined average within the underlying distribution of stimuli.

Both tasks demonstrated a consistent, though partial, central tendency bias. On average, the shift in the PSE did not fully align with the center of the stimulus distribution. However, performance differed between the two tasks. In particular, the vibrotactile task exhibited greater response variability. While the average shift in the tactile PSEs was similar to that observed in the color task, individual responses varied widely—some participants showed a complete shift in their PSE, while others remained relatively stable across the presented ranges. This fits with previous work showing that tactile perception thresholds are highly variable across individuals (Gertz et al., 2017). Notably, we did find differences in discrimination performance between tasks, as these were reflected in the slopes of the psychometric functions (*σ*), as well as in the guess and lapse rates. Specifically, our analyses revealed that the *σ*, guess, and lapse rate values were larger and more variable in the vibrotactile than the color task, indicating worse discrimination performance and greater variability in performance. One possible explanation of this finding is that we chose vibrotactile intensities that were separate by 1.2 JNDs, as these were determined in a previous study that used a similar, but not identical, paradigm (Voudouris and Fiehler, 2017), as the duration of the vibrotactile stimuli was different. In this case, despite our efforts, the separation of the intensities might not have been matched to the two (color and vibrotactile) tasks employed here nor to the perception of our specific participants. Other possibilities might include that noise between the tactile receptors and the perceptual decision stage deteriorates tactile discrimination performance (Arabzadeh et al., 2014) or that the propagation of our 750-ms-long vibrations across the stimulated skin (Shao et al., 2016) might activate multiple receptors and diminish the precision of the tactile estimate. However, these possibilities do not preclude our main question in this study, namely, whether perception of vibrotactile intensity is influenced by the underlying stimulus distribution.

What are the origins of this central tendency bias? Previous work has proposed a Bayesian framework to explain this phenomenon. The Bayesian framework is a statistical approach where prior knowledge and the context in which a stimulus is presented are factored into decision-making. Jazayeri and Shadlen (Jazayeri and Shadlen, 2010) proposed that participants use this Bayesian strategy to reduce uncertainty when making decisions. This bias is also known as the range effect. Hollingworth (Hollingworth, 1910) and Parducci (Parducci, 1965) suggested the perception of a stimulus depends on the range of stimuli presented. As a result, the perceived range may become compressed, leading to a preference for the central stimulus in the distribution.

Previous cognitive and behavioral studies indicated that visual and vibrotactile perception share common mechanisms (Amedi, 2002; Lee Masson et al., 2016; Sathian and Lacey, 2022). Some brain areas involved in processing visual stimuli are also engaged in processing vibrotactile and haptic stimuli (Feinberg et al., 1986). Given that a central tendency bias was observed in both color and vibrotactile discrimination tasks, it raises the question of whether these effects arise from shared underlying mechanisms. If such mechanisms are indeed shared, we might expect individual differences to manifest similarly across both sensory modalities, which would be in line with previous evidence (Yarbus, 1967; Zhang and Alais, 2020; Kondo et al., 2022; Chow et al., 2023), our study was not designed to directly test it. One might predict that individuals who exhibit a stronger central tendency bias in the color task would also show a greater bias in the vibrotactile task, reflecting shared mechanisms and individual differences. However, our findings do not provide evidence for this, as we did not find a correlation between the magnitude of the central tendency bias in color and vibrotactile discrimination. Yet, it is important to mention that, as our study was not designed to test this question, our sample size is about one-third of the size required to find a small effect size. Studies on individual differences typically include >60 participants (Buswell, 1935; De Haas et al., 2019). Future research investigating individual differences and shared mechanisms in the central tendency effect across modalities should use larger sample sizes to increase statistical power.

In short, our study replicates the well-established central tendency bias effect in color perception while further demonstrating, for the first time, that such biases also arise when discriminating the intensity of vibrotactile stimulation. This is important to consider in various domains, such as when interpreting tactile detection and discrimination thresholds across different studies and when deciding what type of tactile stimulations should be used when designing tactile interfaces.

## Synthesis

Reviewing Editor: Ifat Levy, Yale School of Medicine Decisions are customarily a result of the Reviewing Editor and the peer reviewers coming together and discussing their recommendations until a consensus is reached. When revisions are invited, a fact-based synthesis statement explaining their decision and outlining what is needed to prepare a revision will be listed below. The following reviewer(s) agreed to reveal their identity: Michael Grubb.

This study demonstrates a central tendency bias in vibrotactile perception, in a small group of participants. This bias has been shown before in visual perception but has not been studied in vibrotactile perception, which makes the findings interesting and important. A strength of the paper is that the authors also employed a standard visual color discrimination task, replicating the bias in the visual domain. Overall, the results suggest that the central tendency bias is common across sensory modalities and will likely inspire follow-up research.

The paper is well written, the analyses are sound and well explained, and the outcomes support the authors' claims. Reviewers had several comments, however, which should be addressed, as detailed below.

- Please remove the unity line from Figure 6 and replace it with a regression line. The point of a unity line in a scatterplot is typically to show where the points fall relative to that line so that one can make a case for one value on the x being different from the value on the y (i.e., all the points are above the unity line). But here, the point seems to be about the correlation between the two variables, so a line of best fit would seem more appropriate.

- The research used psignifit 4 toolbox in MATLAB and JASP. Could you present the parameters generated by the toolbox?

- The authors acknowledge that their small sample limits the power to do a full individual differences assessment. However, they may have more sensitivity to detect such differences, if they fit the data jointly rather than separately, minimizing noise that arises from estimating the parameters separately. The authors are encouraged to provide additional analysis, fitting a standard logistic or probit model with joint data and report the correlations for the bias and the choice accuracy (or inverse temperature). Given that they have >1000 trials in total from the two tasks, it's conceivable to fit a probit regression model and derive/estimate 12 parameters (6 bias and 6 slope parameters). Moreover, it is also reasonable to use a common scale (1-5) for both color and vibrotactile tasks when jointly fitting the data with the probit.

- It will be helpful if the counterfactual of the central tendency bias is explained, as a way of giving the reader a better intuition of what's at stake with the manuscript. Is it reasonable to believe that the counterfactual hypothesis to the central tendency bias is that there is no shift?

- Line 313: what does standard hues/vibrotactile stimuli mean? Is this the standard deviation? Please explain.

- Figure 3: the shades of yellow-green and blue-green are almost indistinguishable. Could you make it more salient for the reader?

- In Fig. 4, the standard error of the mean only has one bar. Are the other bars smaller than the data points?

- There is a Supplementary Analysis that (i.e., on sigma) that is being discussed during the Discussion. The flow of the paper may be better if this is incorporated in the Results section.
