# Supplementary figures and images for: Stimulus Distribution Shapes Color and Vibrotactile Perception
Source: eNeuro. 2025 Oct 10;12(10):ENEURO.0121-25.2025. doi: 10.1523/ENEURO.0121-25.2025 (PMC12517767; doi:10.1523/ENEURO.0121-25.2025)

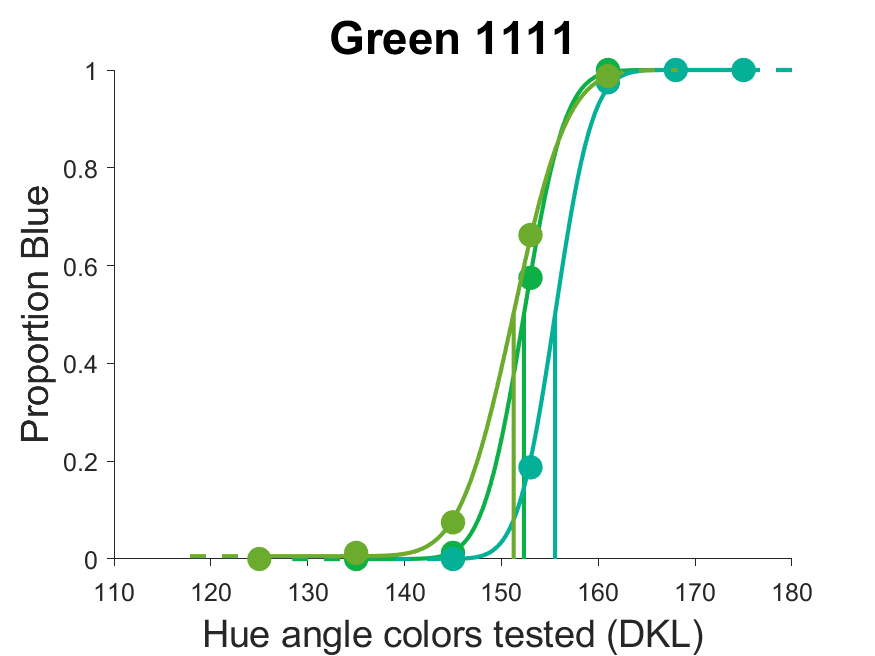

Supplement: Figure 5-1 — Guess and lapse rates for the color and vibrotactile tasks. Panels A and B show the guess rates for the color and vibrotactile tasks, respectively, while panels C and D show the lapse rates for the color and vibrotactile tasks. Download Figure 5-1, ZIP file. [file eneuro-12-ENEURO.0121-25.2025-s002.zip › PsychometricFits_Color/Psychometric_1111.png]

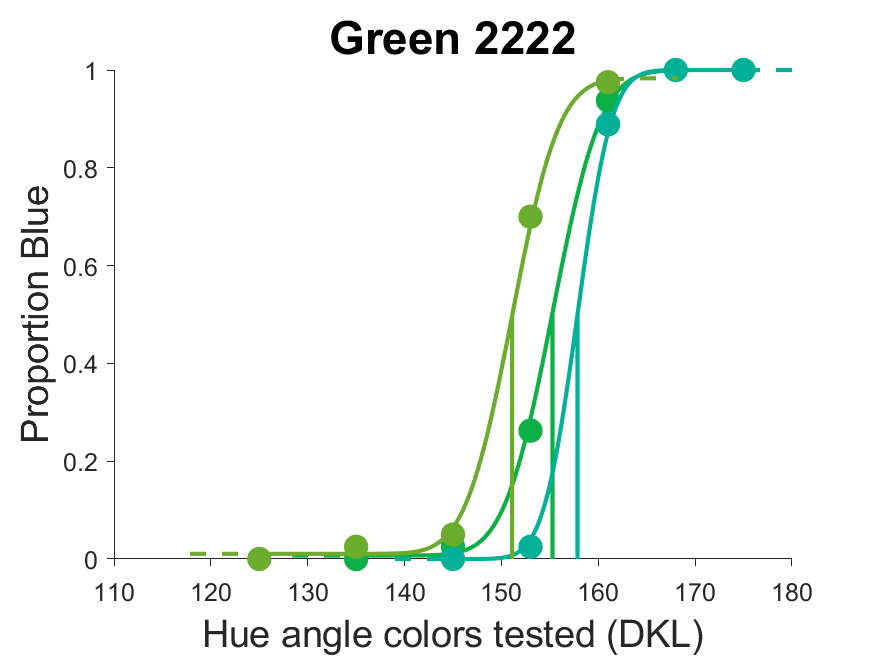

Supplement: Figure 5-1 — Guess and lapse rates for the color and vibrotactile tasks. Panels A and B show the guess rates for the color and vibrotactile tasks, respectively, while panels C and D show the lapse rates for the color and vibrotactile tasks. Download Figure 5-1, ZIP file. [file eneuro-12-ENEURO.0121-25.2025-s002.zip › PsychometricFits_Color/Psychometric_2222.png]

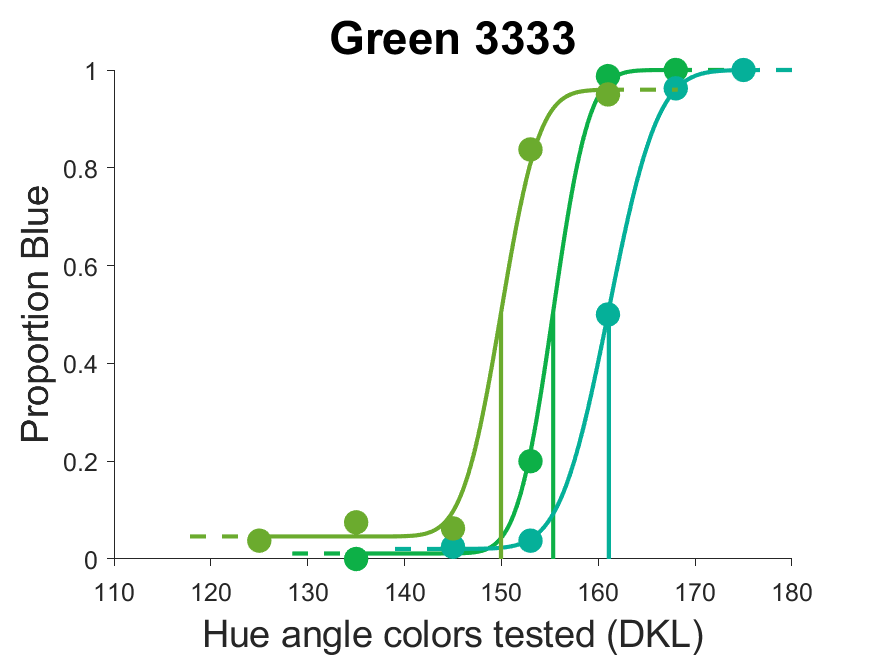

Supplement: Figure 5-1 — Guess and lapse rates for the color and vibrotactile tasks. Panels A and B show the guess rates for the color and vibrotactile tasks, respectively, while panels C and D show the lapse rates for the color and vibrotactile tasks. Download Figure 5-1, ZIP file. [file eneuro-12-ENEURO.0121-25.2025-s002.zip › PsychometricFits_Color/Psychometric_3333.png]

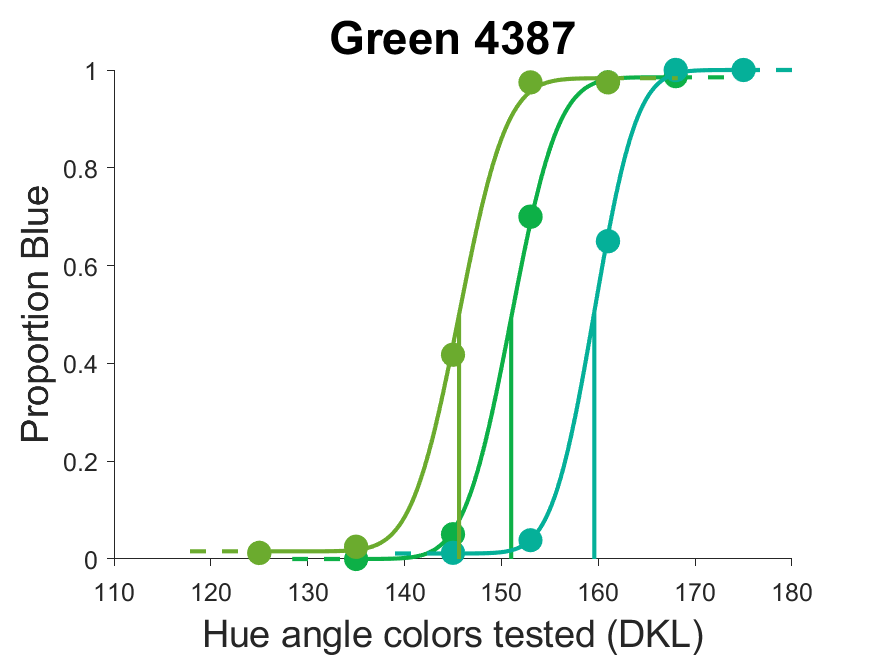

Supplement: Figure 5-1 — Guess and lapse rates for the color and vibrotactile tasks. Panels A and B show the guess rates for the color and vibrotactile tasks, respectively, while panels C and D show the lapse rates for the color and vibrotactile tasks. Download Figure 5-1, ZIP file. [file eneuro-12-ENEURO.0121-25.2025-s002.zip › PsychometricFits_Color/Psychometric_4387.png]

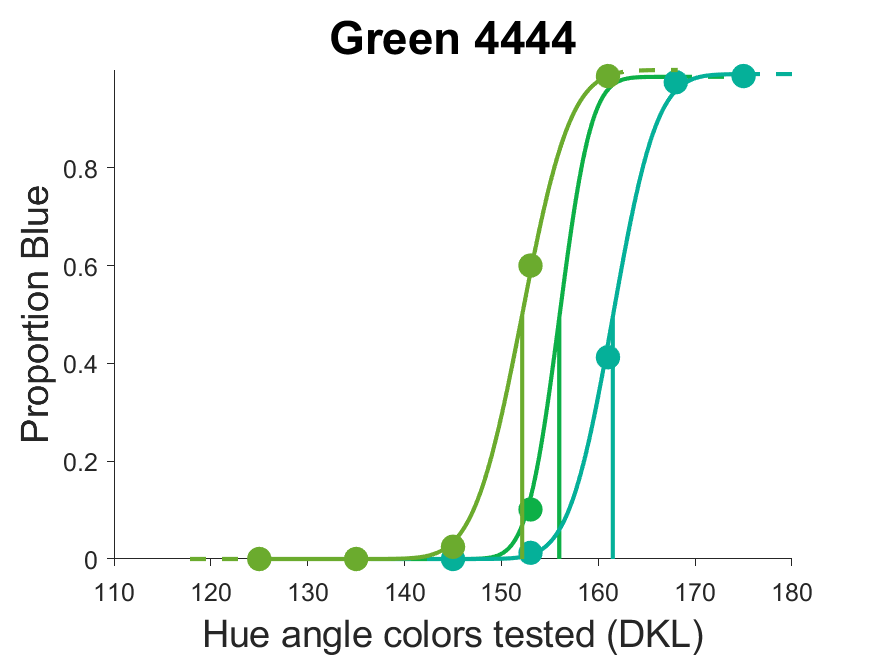

Supplement: Figure 5-1 — Guess and lapse rates for the color and vibrotactile tasks. Panels A and B show the guess rates for the color and vibrotactile tasks, respectively, while panels C and D show the lapse rates for the color and vibrotactile tasks. Download Figure 5-1, ZIP file. [file eneuro-12-ENEURO.0121-25.2025-s002.zip › PsychometricFits_Color/Psychometric_4444.png]

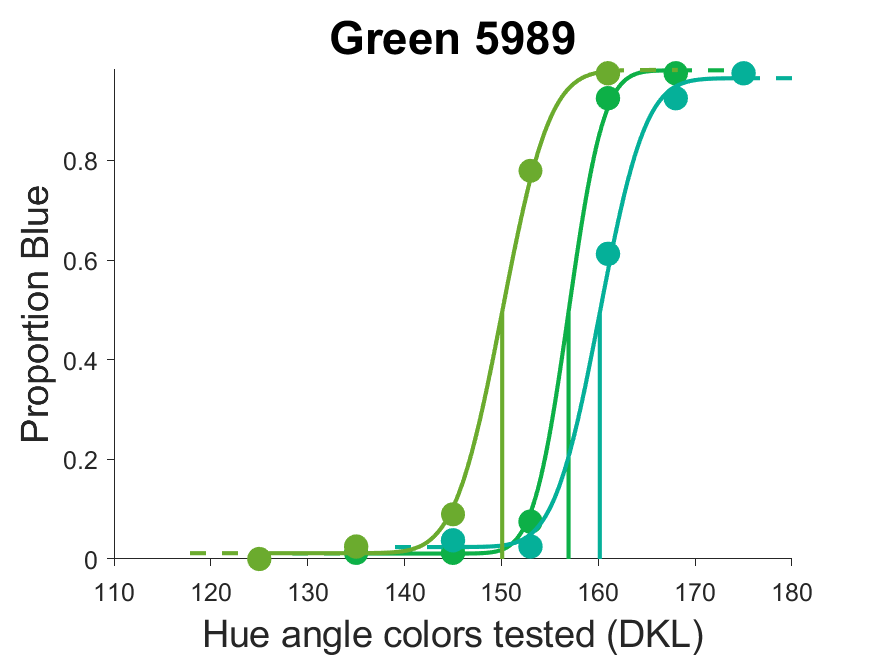

Supplement: Figure 5-1 — Guess and lapse rates for the color and vibrotactile tasks. Panels A and B show the guess rates for the color and vibrotactile tasks, respectively, while panels C and D show the lapse rates for the color and vibrotactile tasks. Download Figure 5-1, ZIP file. [file eneuro-12-ENEURO.0121-25.2025-s002.zip › PsychometricFits_Color/Psychometric_5989.png]

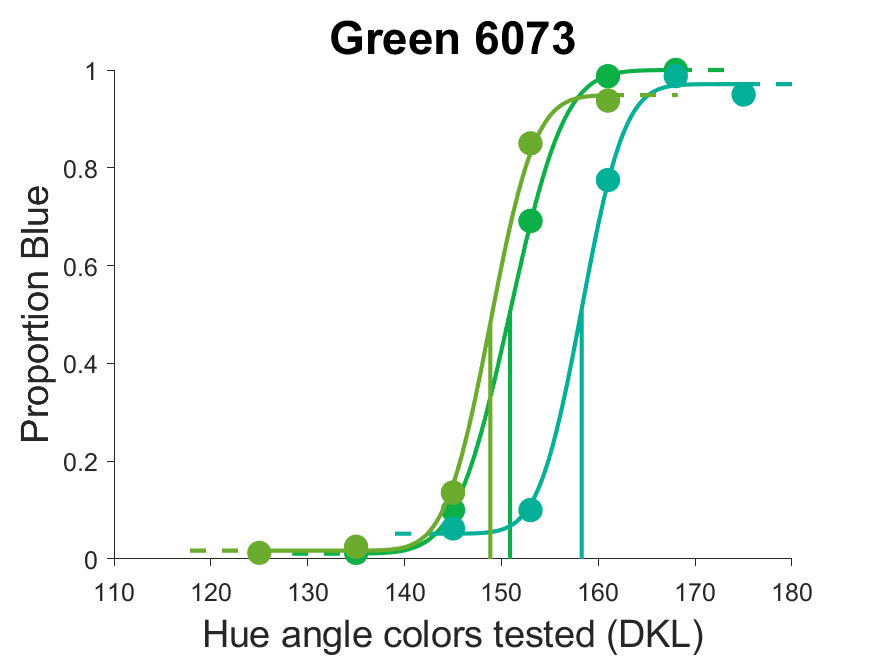

Supplement: Figure 5-1 — Guess and lapse rates for the color and vibrotactile tasks. Panels A and B show the guess rates for the color and vibrotactile tasks, respectively, while panels C and D show the lapse rates for the color and vibrotactile tasks. Download Figure 5-1, ZIP file. [file eneuro-12-ENEURO.0121-25.2025-s002.zip › PsychometricFits_Color/Psychometric_6073.png]

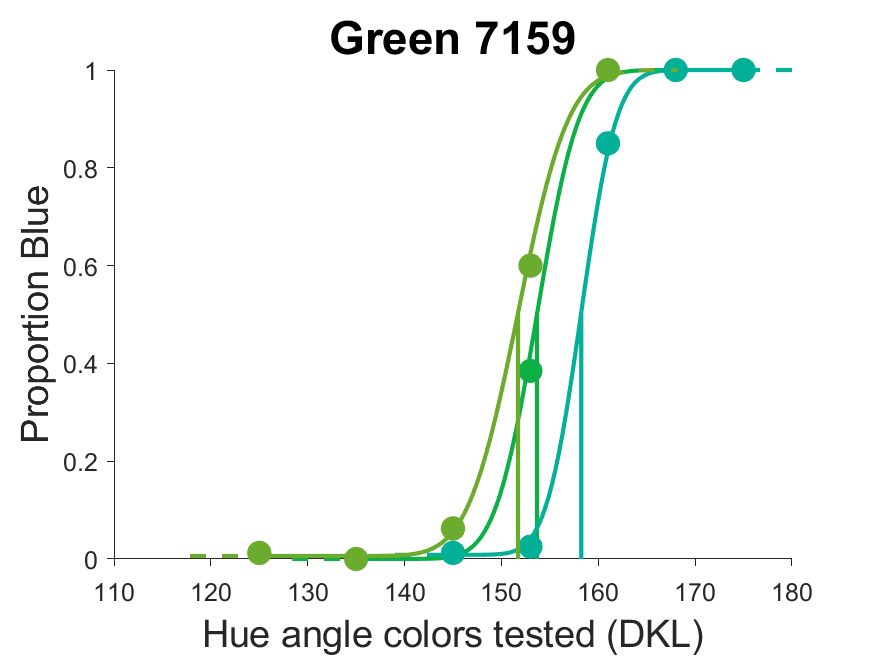

Supplement: Figure 5-1 — Guess and lapse rates for the color and vibrotactile tasks. Panels A and B show the guess rates for the color and vibrotactile tasks, respectively, while panels C and D show the lapse rates for the color and vibrotactile tasks. Download Figure 5-1, ZIP file. [file eneuro-12-ENEURO.0121-25.2025-s002.zip › PsychometricFits_Color/Psychometric_7159.png]

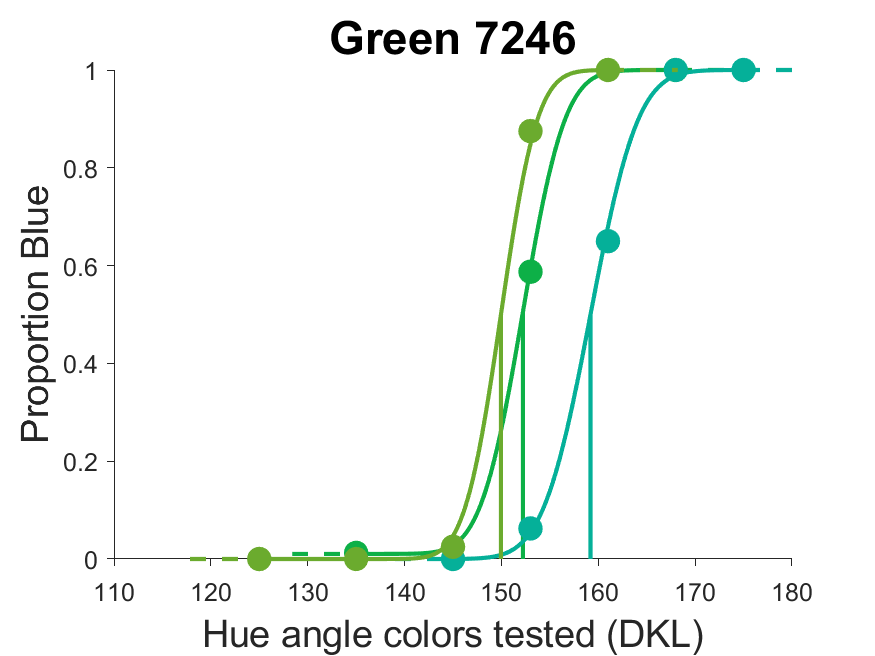

Supplement: Figure 5-1 — Guess and lapse rates for the color and vibrotactile tasks. Panels A and B show the guess rates for the color and vibrotactile tasks, respectively, while panels C and D show the lapse rates for the color and vibrotactile tasks. Download Figure 5-1, ZIP file. [file eneuro-12-ENEURO.0121-25.2025-s002.zip › PsychometricFits_Color/Psychometric_7246.png]

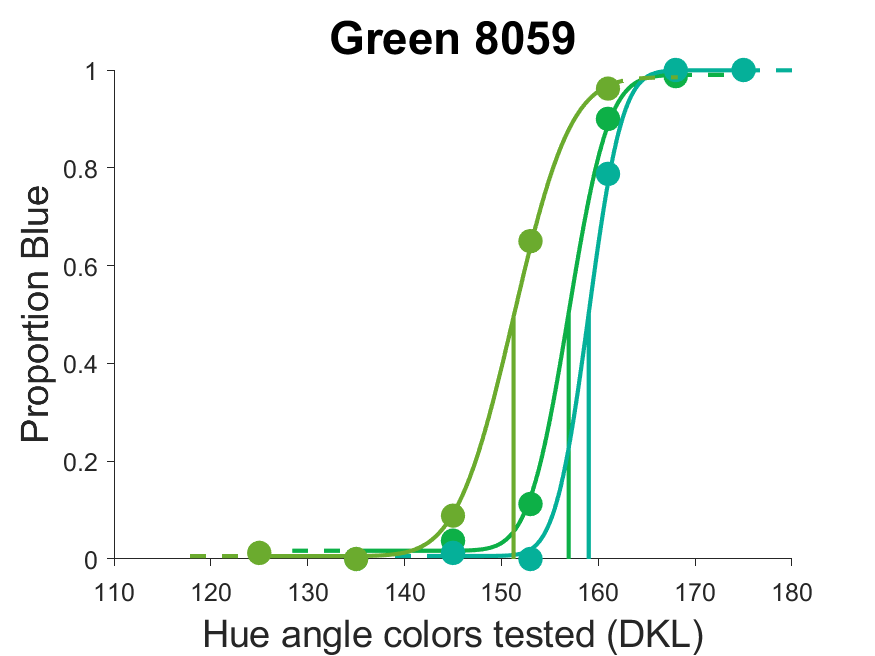

Supplement: Figure 5-1 — Guess and lapse rates for the color and vibrotactile tasks. Panels A and B show the guess rates for the color and vibrotactile tasks, respectively, while panels C and D show the lapse rates for the color and vibrotactile tasks. Download Figure 5-1, ZIP file. [file eneuro-12-ENEURO.0121-25.2025-s002.zip › PsychometricFits_Color/Psychometric_8059.png]

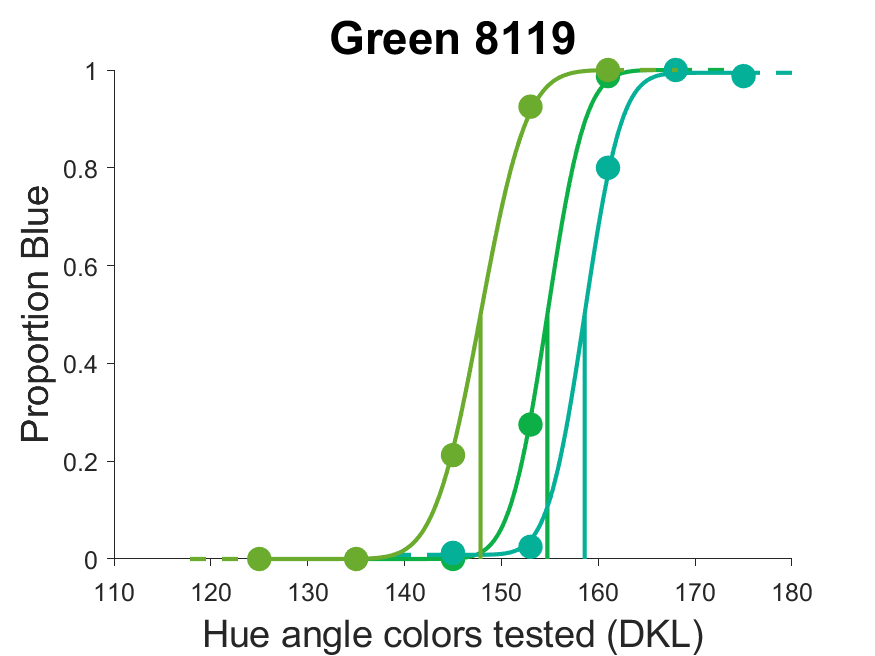

Supplement: Figure 5-1 — Guess and lapse rates for the color and vibrotactile tasks. Panels A and B show the guess rates for the color and vibrotactile tasks, respectively, while panels C and D show the lapse rates for the color and vibrotactile tasks. Download Figure 5-1, ZIP file. [file eneuro-12-ENEURO.0121-25.2025-s002.zip › PsychometricFits_Color/Psychometric_8119.png]

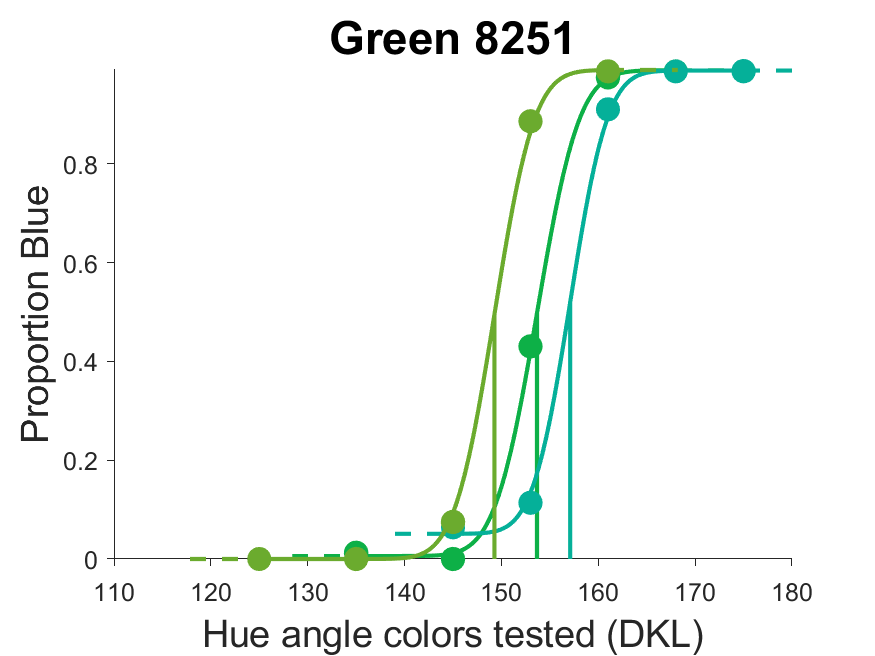

Supplement: Figure 5-1 — Guess and lapse rates for the color and vibrotactile tasks. Panels A and B show the guess rates for the color and vibrotactile tasks, respectively, while panels C and D show the lapse rates for the color and vibrotactile tasks. Download Figure 5-1, ZIP file. [file eneuro-12-ENEURO.0121-25.2025-s002.zip › PsychometricFits_Color/Psychometric_8251.png]

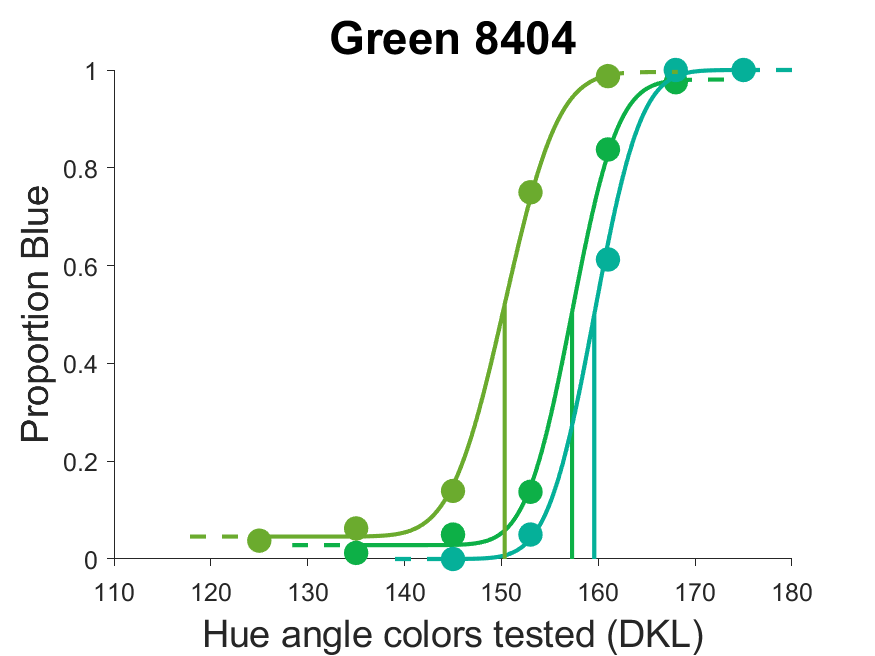

Supplement: Figure 5-1 — Guess and lapse rates for the color and vibrotactile tasks. Panels A and B show the guess rates for the color and vibrotactile tasks, respectively, while panels C and D show the lapse rates for the color and vibrotactile tasks. Download Figure 5-1, ZIP file. [file eneuro-12-ENEURO.0121-25.2025-s002.zip › PsychometricFits_Color/Psychometric_8404.png]

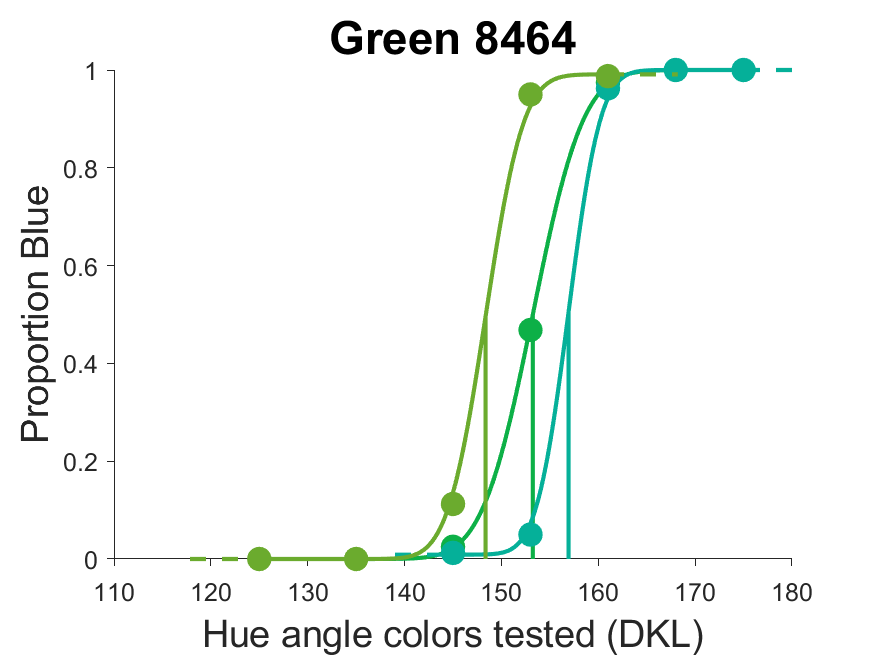

Supplement: Figure 5-1 — Guess and lapse rates for the color and vibrotactile tasks. Panels A and B show the guess rates for the color and vibrotactile tasks, respectively, while panels C and D show the lapse rates for the color and vibrotactile tasks. Download Figure 5-1, ZIP file. [file eneuro-12-ENEURO.0121-25.2025-s002.zip › PsychometricFits_Color/Psychometric_8464.png]

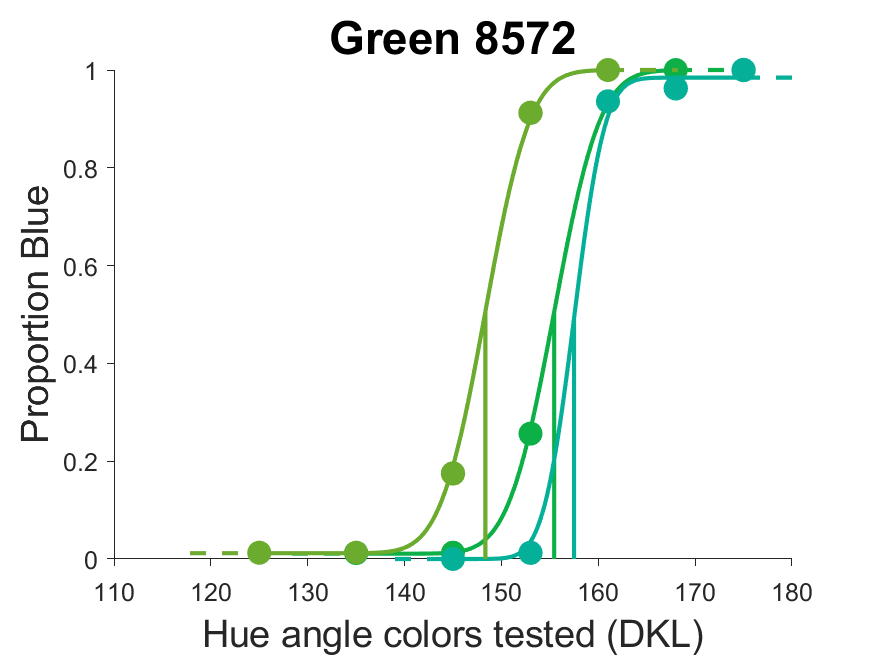

Supplement: Figure 5-1 — Guess and lapse rates for the color and vibrotactile tasks. Panels A and B show the guess rates for the color and vibrotactile tasks, respectively, while panels C and D show the lapse rates for the color and vibrotactile tasks. Download Figure 5-1, ZIP file. [file eneuro-12-ENEURO.0121-25.2025-s002.zip › PsychometricFits_Color/Psychometric_8572.png]

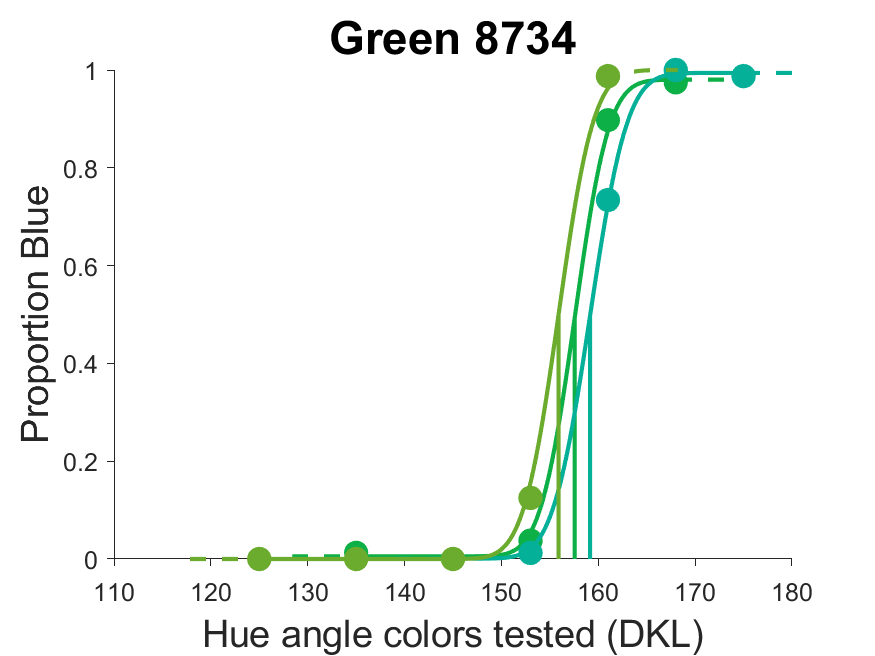

Supplement: Figure 5-1 — Guess and lapse rates for the color and vibrotactile tasks. Panels A and B show the guess rates for the color and vibrotactile tasks, respectively, while panels C and D show the lapse rates for the color and vibrotactile tasks. Download Figure 5-1, ZIP file. [file eneuro-12-ENEURO.0121-25.2025-s002.zip › PsychometricFits_Color/Psychometric_8734.png]

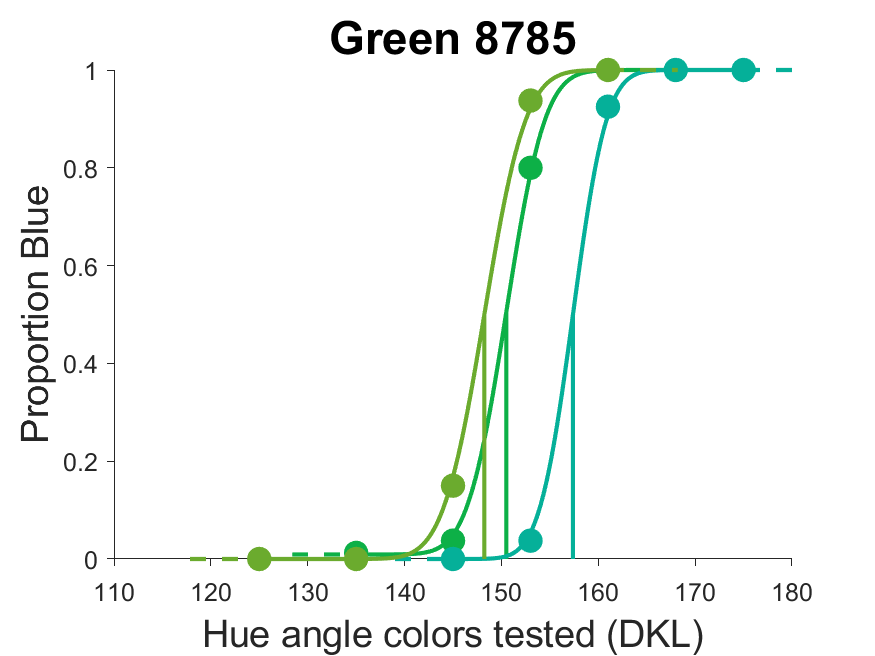

Supplement: Figure 5-1 — Guess and lapse rates for the color and vibrotactile tasks. Panels A and B show the guess rates for the color and vibrotactile tasks, respectively, while panels C and D show the lapse rates for the color and vibrotactile tasks. Download Figure 5-1, ZIP file. [file eneuro-12-ENEURO.0121-25.2025-s002.zip › PsychometricFits_Color/Psychometric_8785.png]

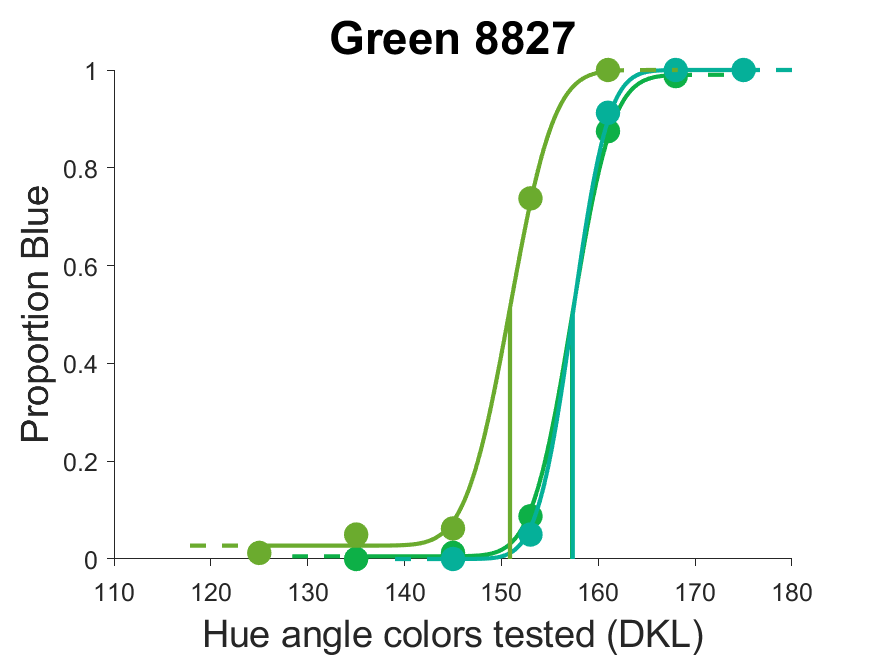

Supplement: Figure 5-1 — Guess and lapse rates for the color and vibrotactile tasks. Panels A and B show the guess rates for the color and vibrotactile tasks, respectively, while panels C and D show the lapse rates for the color and vibrotactile tasks. Download Figure 5-1, ZIP file. [file eneuro-12-ENEURO.0121-25.2025-s002.zip › PsychometricFits_Color/Psychometric_8827.png]

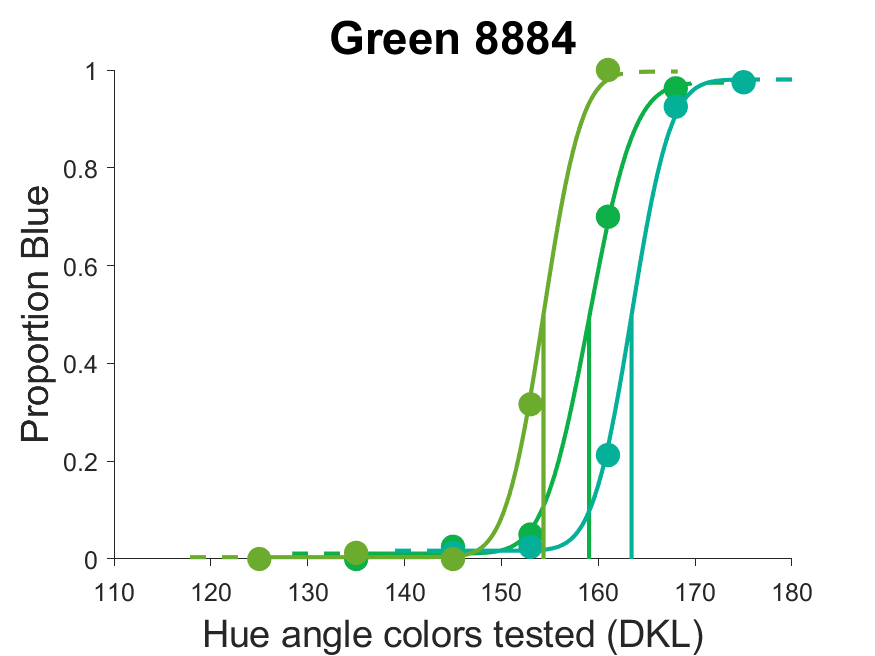

Supplement: Figure 5-1 — Guess and lapse rates for the color and vibrotactile tasks. Panels A and B show the guess rates for the color and vibrotactile tasks, respectively, while panels C and D show the lapse rates for the color and vibrotactile tasks. Download Figure 5-1, ZIP file. [file eneuro-12-ENEURO.0121-25.2025-s002.zip › PsychometricFits_Color/Psychometric_8884.png]

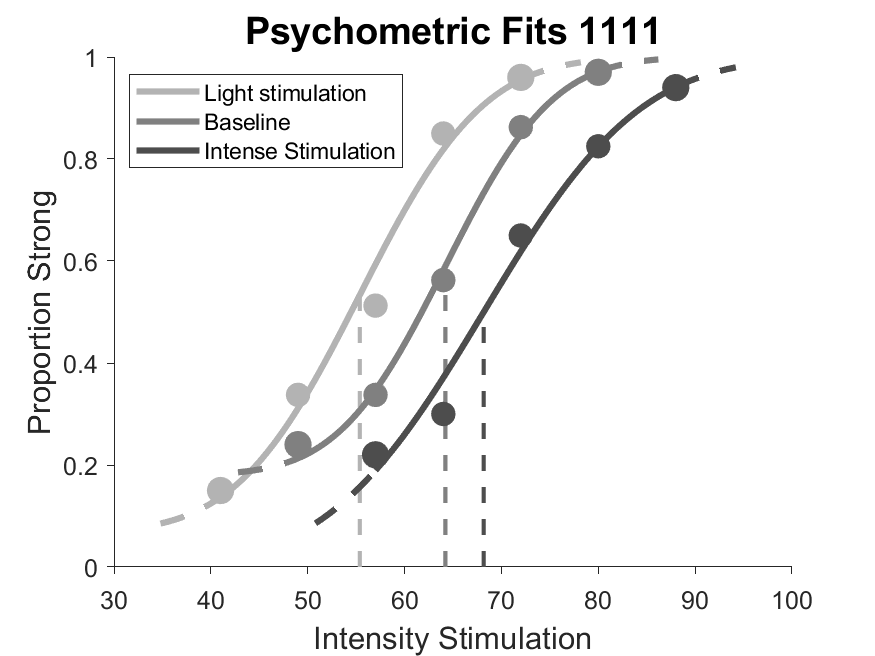

Supplement: Figure 5-1 — Guess and lapse rates for the color and vibrotactile tasks. Panels A and B show the guess rates for the color and vibrotactile tasks, respectively, while panels C and D show the lapse rates for the color and vibrotactile tasks. Download Figure 5-1, ZIP file. [file eneuro-12-ENEURO.0121-25.2025-s002.zip › PsychometricFits_Vibrotactile/Psychometric_1111.png]

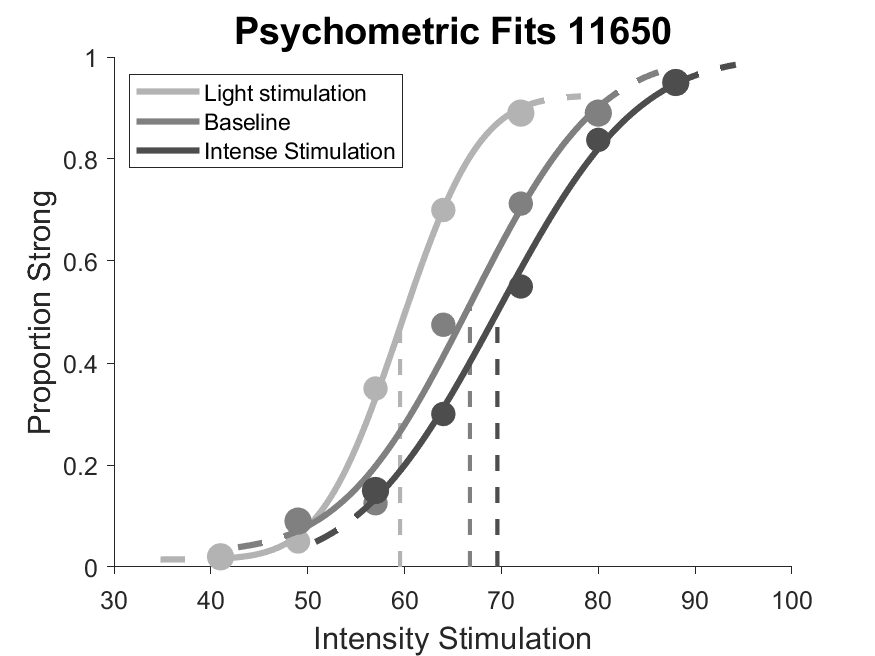

Supplement: Figure 5-1 — Guess and lapse rates for the color and vibrotactile tasks. Panels A and B show the guess rates for the color and vibrotactile tasks, respectively, while panels C and D show the lapse rates for the color and vibrotactile tasks. Download Figure 5-1, ZIP file. [file eneuro-12-ENEURO.0121-25.2025-s002.zip › PsychometricFits_Vibrotactile/Psychometric_11650.png]

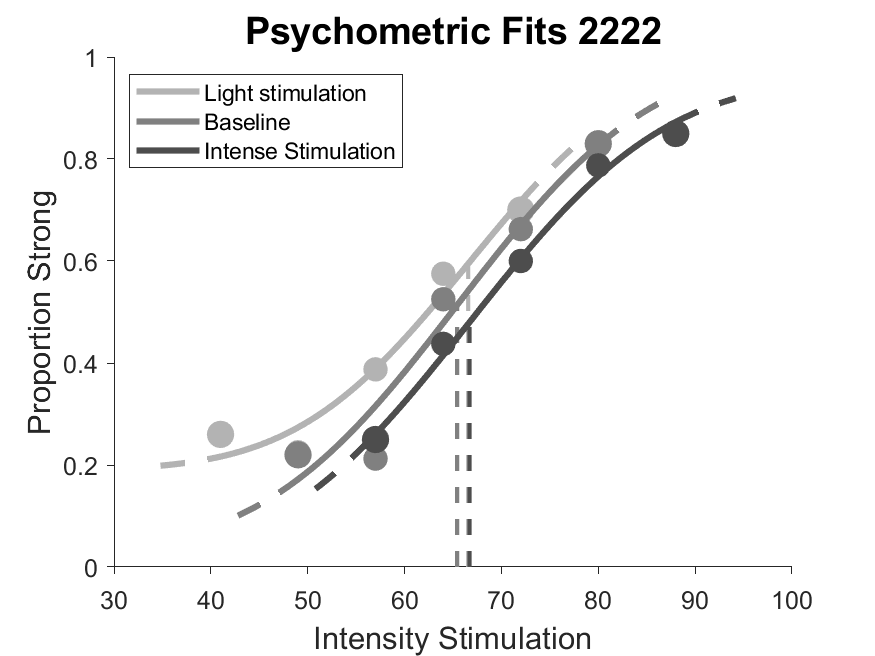

Supplement: Figure 5-1 — Guess and lapse rates for the color and vibrotactile tasks. Panels A and B show the guess rates for the color and vibrotactile tasks, respectively, while panels C and D show the lapse rates for the color and vibrotactile tasks. Download Figure 5-1, ZIP file. [file eneuro-12-ENEURO.0121-25.2025-s002.zip › PsychometricFits_Vibrotactile/Psychometric_2222.png]

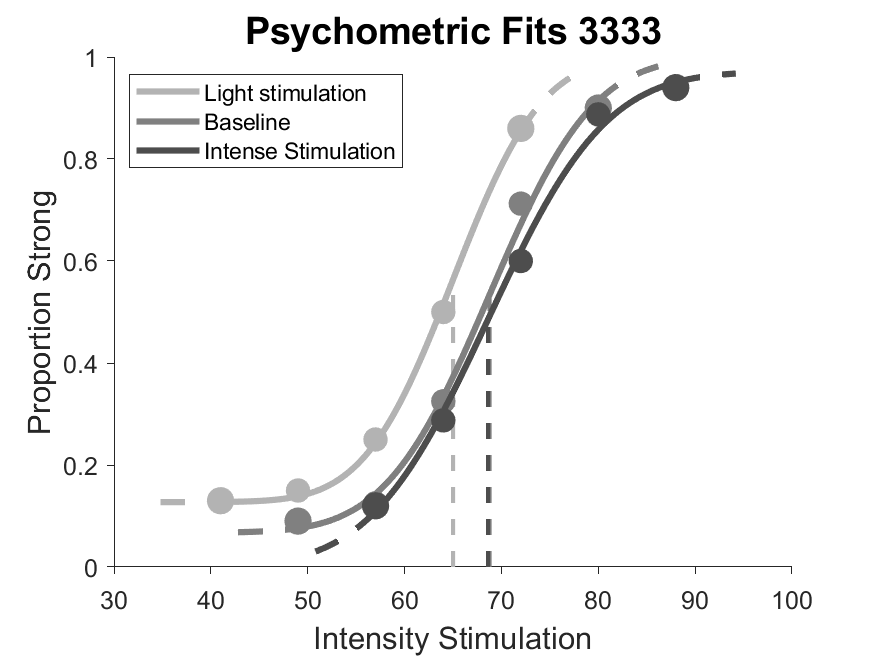

Supplement: Figure 5-1 — Guess and lapse rates for the color and vibrotactile tasks. Panels A and B show the guess rates for the color and vibrotactile tasks, respectively, while panels C and D show the lapse rates for the color and vibrotactile tasks. Download Figure 5-1, ZIP file. [file eneuro-12-ENEURO.0121-25.2025-s002.zip › PsychometricFits_Vibrotactile/Psychometric_3333.png]

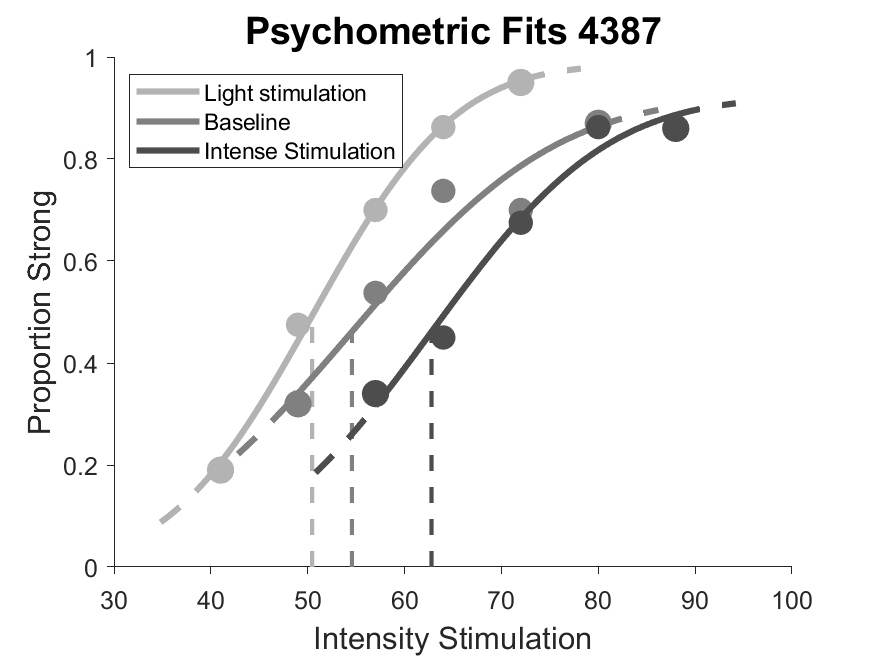

Supplement: Figure 5-1 — Guess and lapse rates for the color and vibrotactile tasks. Panels A and B show the guess rates for the color and vibrotactile tasks, respectively, while panels C and D show the lapse rates for the color and vibrotactile tasks. Download Figure 5-1, ZIP file. [file eneuro-12-ENEURO.0121-25.2025-s002.zip › PsychometricFits_Vibrotactile/Psychometric_4387.png]

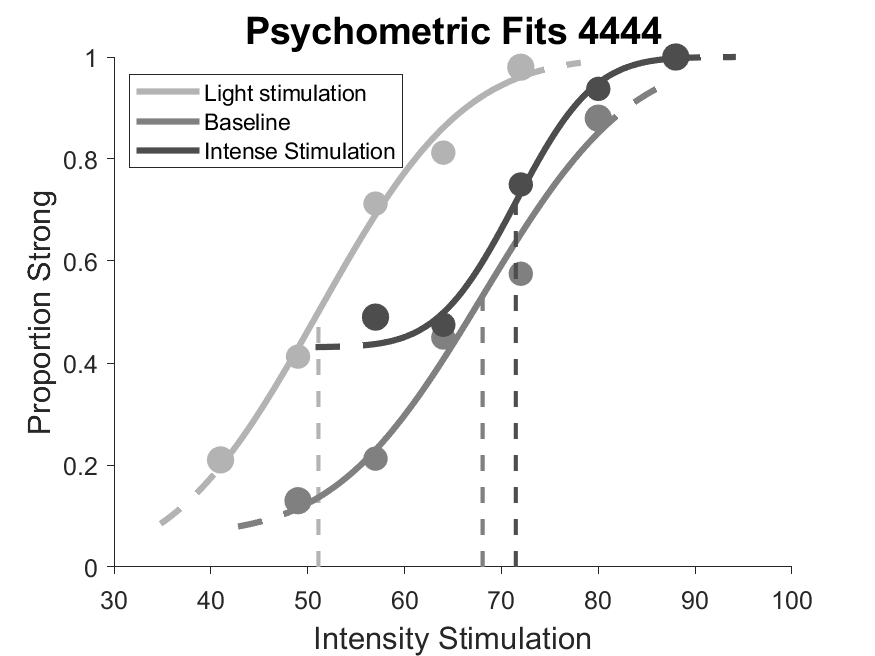

Supplement: Figure 5-1 — Guess and lapse rates for the color and vibrotactile tasks. Panels A and B show the guess rates for the color and vibrotactile tasks, respectively, while panels C and D show the lapse rates for the color and vibrotactile tasks. Download Figure 5-1, ZIP file. [file eneuro-12-ENEURO.0121-25.2025-s002.zip › PsychometricFits_Vibrotactile/Psychometric_4444.png]

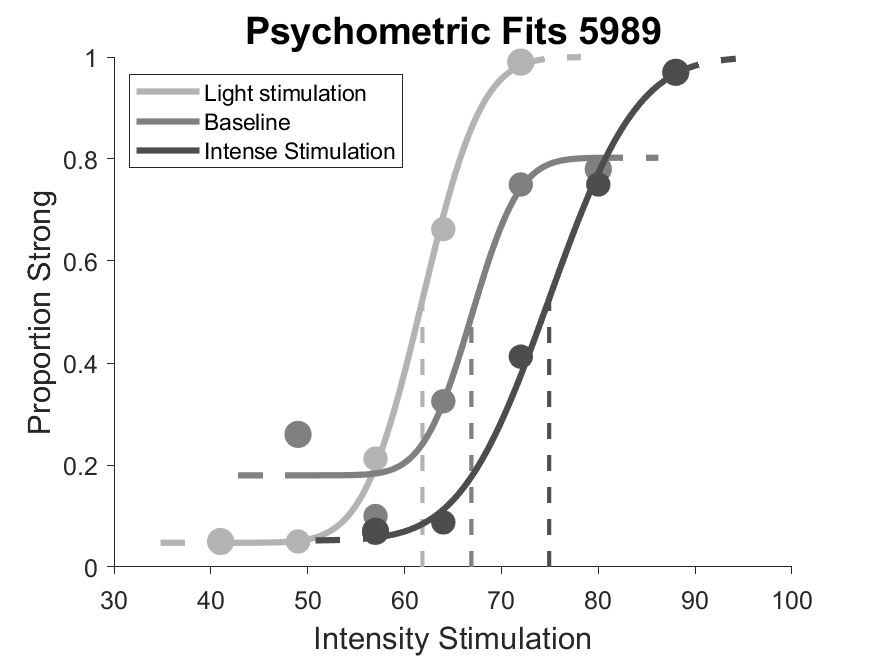

Supplement: Figure 5-1 — Guess and lapse rates for the color and vibrotactile tasks. Panels A and B show the guess rates for the color and vibrotactile tasks, respectively, while panels C and D show the lapse rates for the color and vibrotactile tasks. Download Figure 5-1, ZIP file. [file eneuro-12-ENEURO.0121-25.2025-s002.zip › PsychometricFits_Vibrotactile/Psychometric_5989.png]

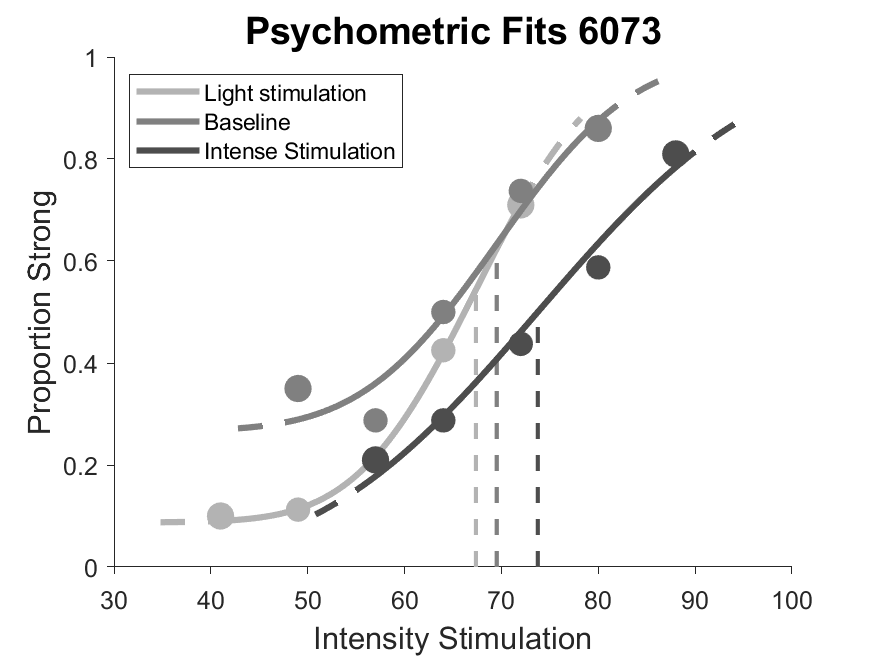

Supplement: Figure 5-1 — Guess and lapse rates for the color and vibrotactile tasks. Panels A and B show the guess rates for the color and vibrotactile tasks, respectively, while panels C and D show the lapse rates for the color and vibrotactile tasks. Download Figure 5-1, ZIP file. [file eneuro-12-ENEURO.0121-25.2025-s002.zip › PsychometricFits_Vibrotactile/Psychometric_6073.png]

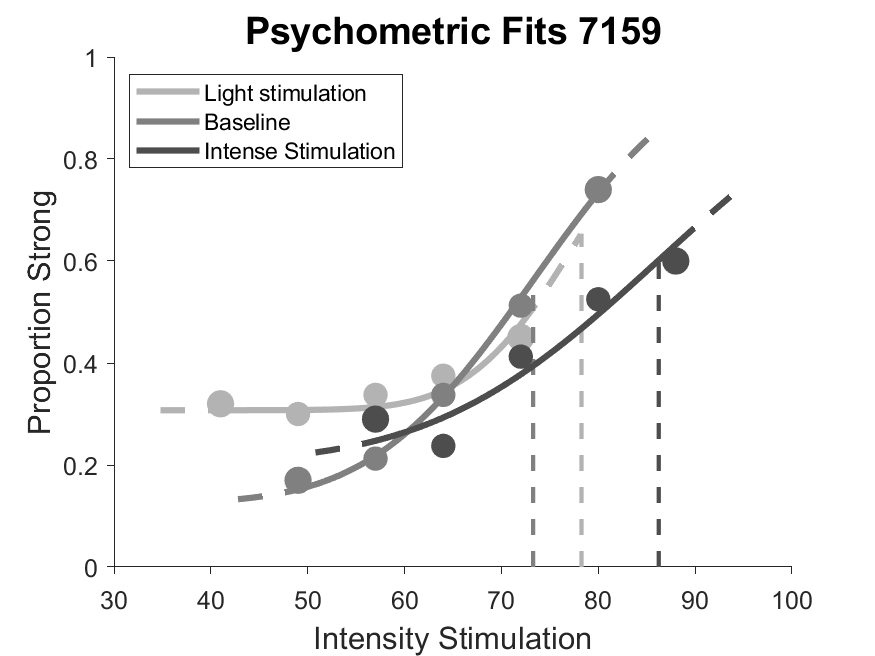

Supplement: Figure 5-1 — Guess and lapse rates for the color and vibrotactile tasks. Panels A and B show the guess rates for the color and vibrotactile tasks, respectively, while panels C and D show the lapse rates for the color and vibrotactile tasks. Download Figure 5-1, ZIP file. [file eneuro-12-ENEURO.0121-25.2025-s002.zip › PsychometricFits_Vibrotactile/Psychometric_7159.png]

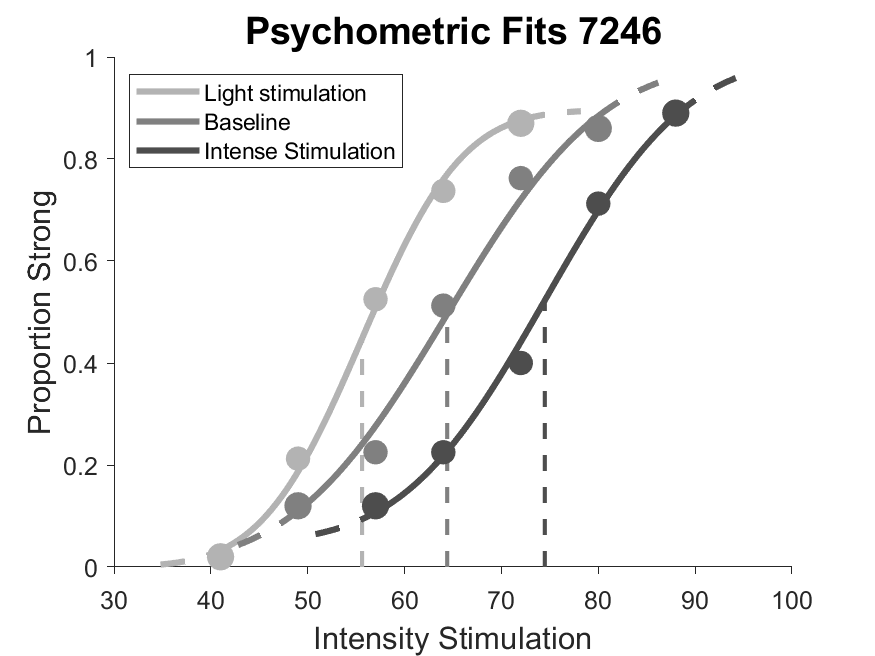

Supplement: Figure 5-1 — Guess and lapse rates for the color and vibrotactile tasks. Panels A and B show the guess rates for the color and vibrotactile tasks, respectively, while panels C and D show the lapse rates for the color and vibrotactile tasks. Download Figure 5-1, ZIP file. [file eneuro-12-ENEURO.0121-25.2025-s002.zip › PsychometricFits_Vibrotactile/Psychometric_7246.png]

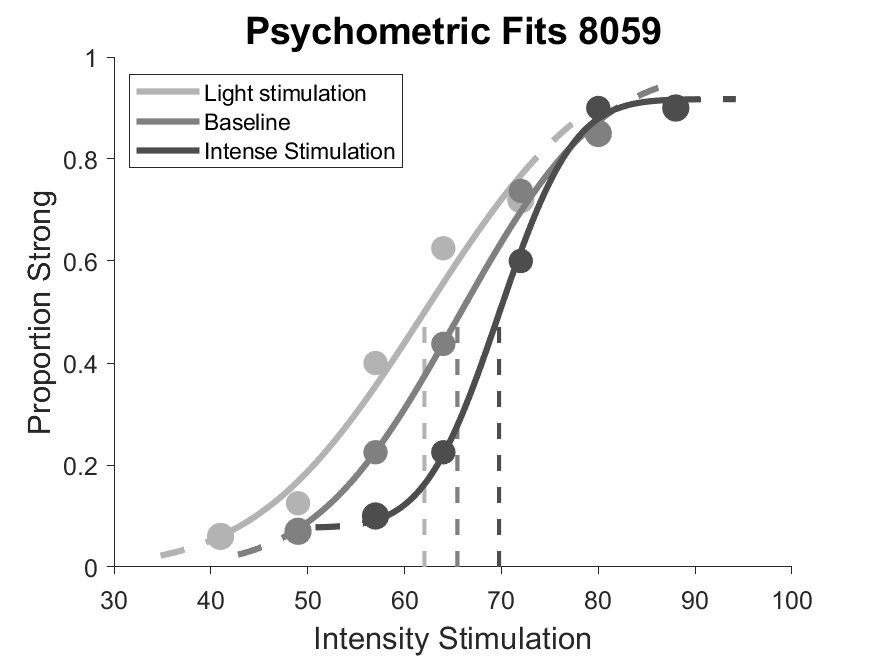

Supplement: Figure 5-1 — Guess and lapse rates for the color and vibrotactile tasks. Panels A and B show the guess rates for the color and vibrotactile tasks, respectively, while panels C and D show the lapse rates for the color and vibrotactile tasks. Download Figure 5-1, ZIP file. [file eneuro-12-ENEURO.0121-25.2025-s002.zip › PsychometricFits_Vibrotactile/Psychometric_8059.png]

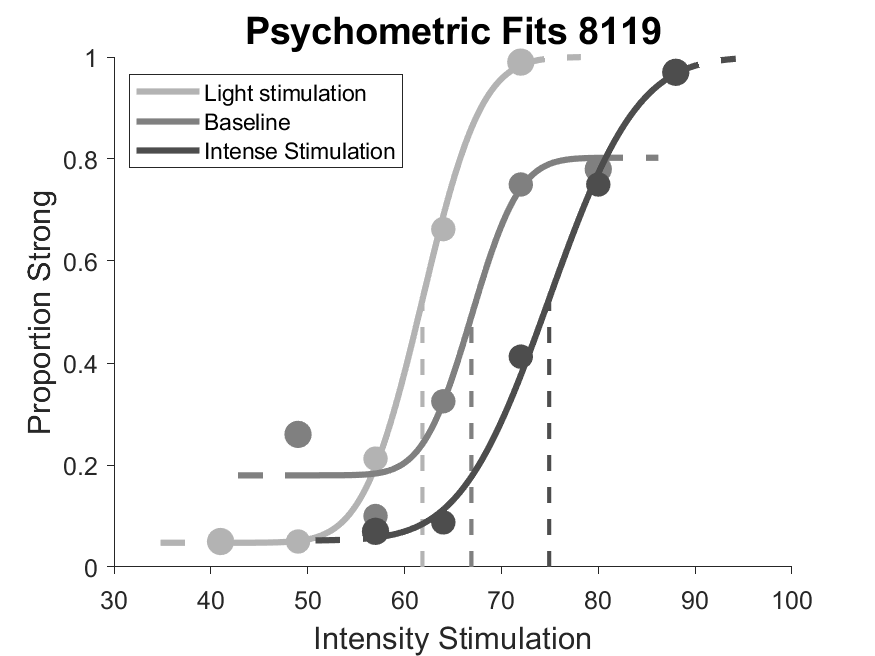

Supplement: Figure 5-1 — Guess and lapse rates for the color and vibrotactile tasks. Panels A and B show the guess rates for the color and vibrotactile tasks, respectively, while panels C and D show the lapse rates for the color and vibrotactile tasks. Download Figure 5-1, ZIP file. [file eneuro-12-ENEURO.0121-25.2025-s002.zip › PsychometricFits_Vibrotactile/Psychometric_8119.png]

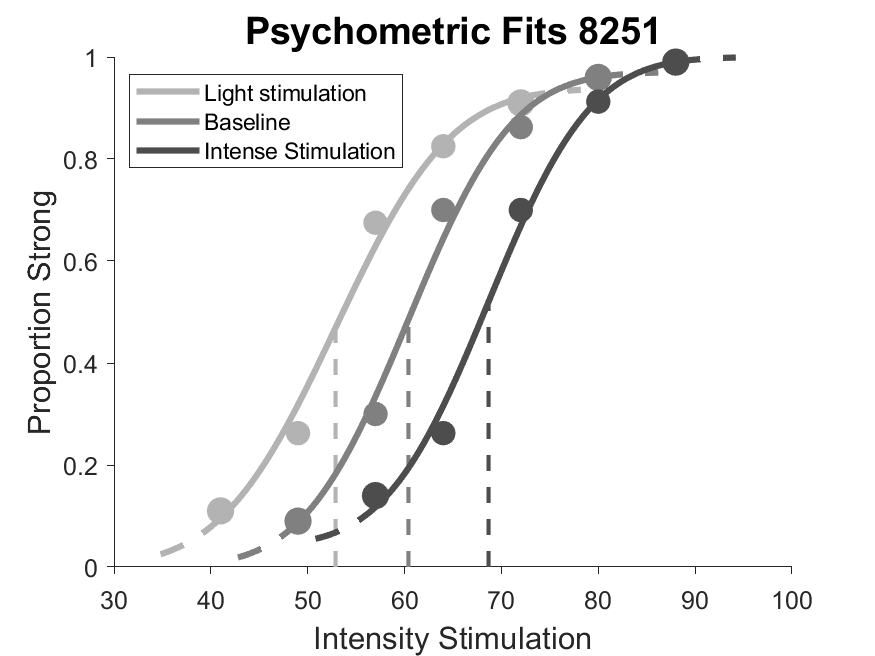

Supplement: Figure 5-1 — Guess and lapse rates for the color and vibrotactile tasks. Panels A and B show the guess rates for the color and vibrotactile tasks, respectively, while panels C and D show the lapse rates for the color and vibrotactile tasks. Download Figure 5-1, ZIP file. [file eneuro-12-ENEURO.0121-25.2025-s002.zip › PsychometricFits_Vibrotactile/Psychometric_8251.png]

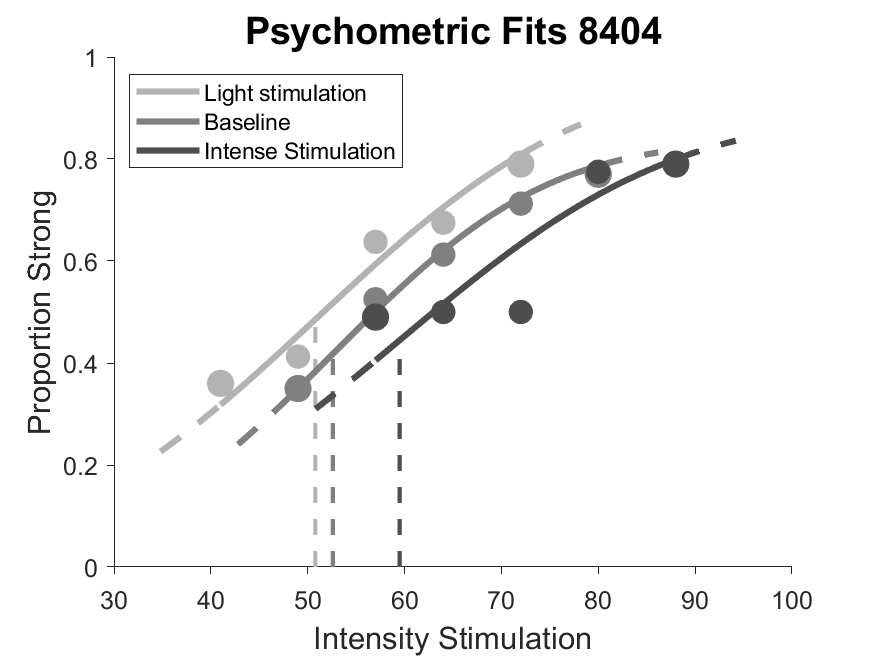

Supplement: Figure 5-1 — Guess and lapse rates for the color and vibrotactile tasks. Panels A and B show the guess rates for the color and vibrotactile tasks, respectively, while panels C and D show the lapse rates for the color and vibrotactile tasks. Download Figure 5-1, ZIP file. [file eneuro-12-ENEURO.0121-25.2025-s002.zip › PsychometricFits_Vibrotactile/Psychometric_8404.png]

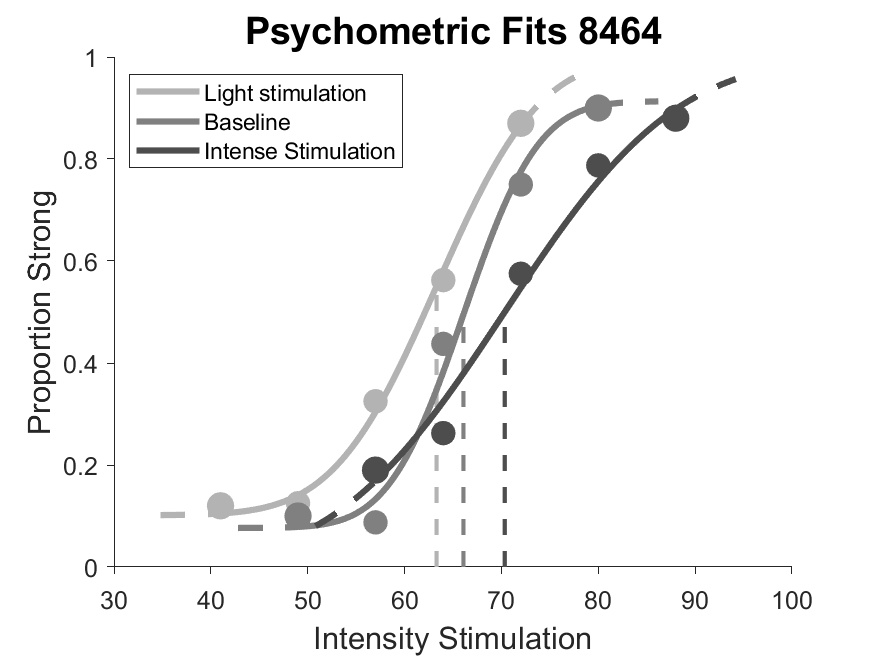

Supplement: Figure 5-1 — Guess and lapse rates for the color and vibrotactile tasks. Panels A and B show the guess rates for the color and vibrotactile tasks, respectively, while panels C and D show the lapse rates for the color and vibrotactile tasks. Download Figure 5-1, ZIP file. [file eneuro-12-ENEURO.0121-25.2025-s002.zip › PsychometricFits_Vibrotactile/Psychometric_8464.png]

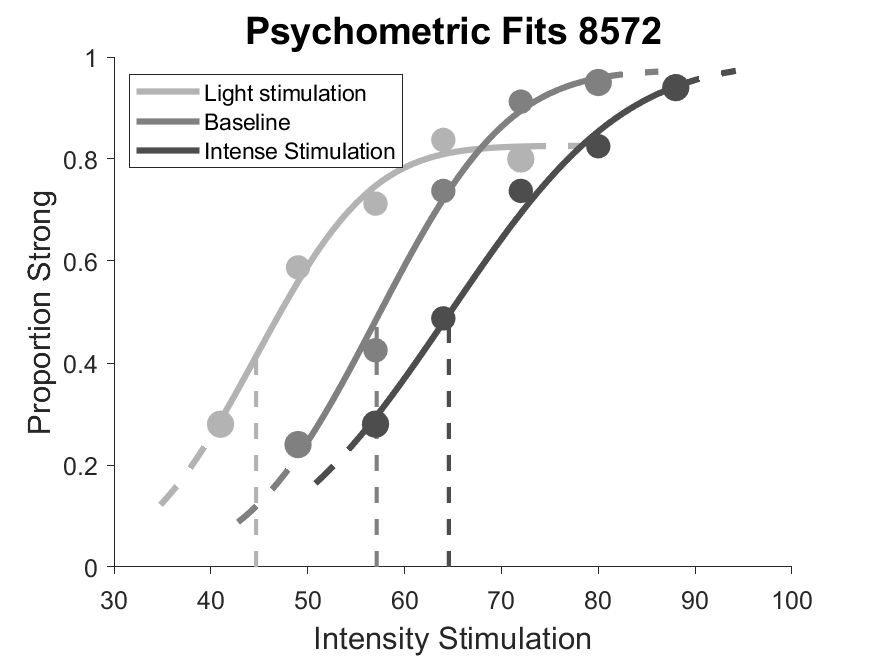

Supplement: Figure 5-1 — Guess and lapse rates for the color and vibrotactile tasks. Panels A and B show the guess rates for the color and vibrotactile tasks, respectively, while panels C and D show the lapse rates for the color and vibrotactile tasks. Download Figure 5-1, ZIP file. [file eneuro-12-ENEURO.0121-25.2025-s002.zip › PsychometricFits_Vibrotactile/Psychometric_8572.png]

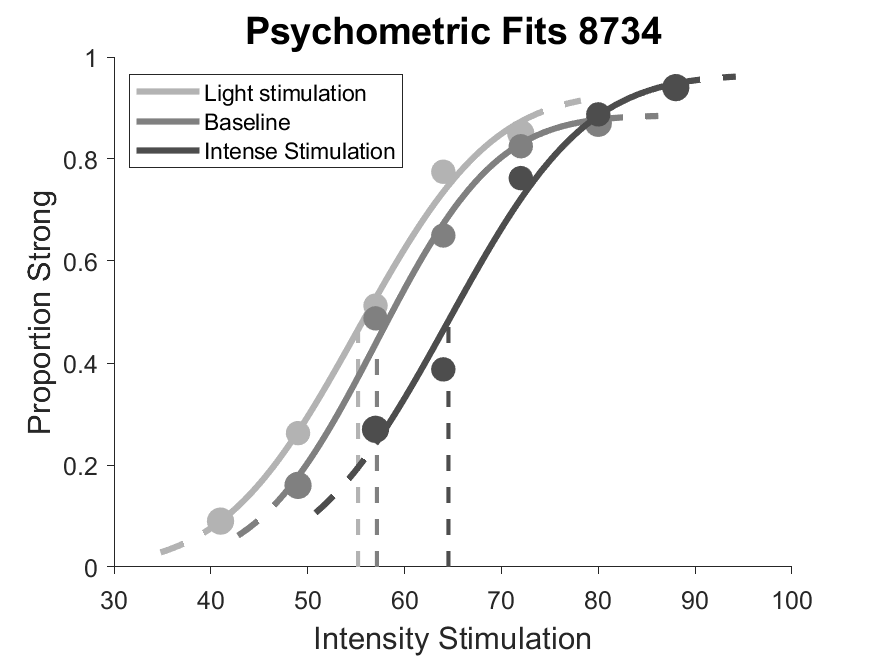

Supplement: Figure 5-1 — Guess and lapse rates for the color and vibrotactile tasks. Panels A and B show the guess rates for the color and vibrotactile tasks, respectively, while panels C and D show the lapse rates for the color and vibrotactile tasks. Download Figure 5-1, ZIP file. [file eneuro-12-ENEURO.0121-25.2025-s002.zip › PsychometricFits_Vibrotactile/Psychometric_8734.png]

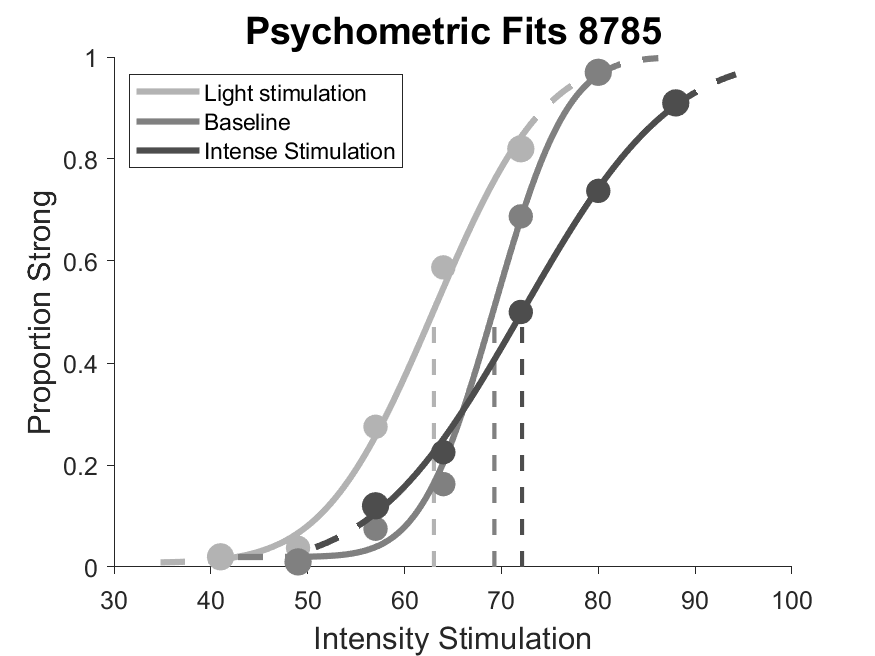

Supplement: Figure 5-1 — Guess and lapse rates for the color and vibrotactile tasks. Panels A and B show the guess rates for the color and vibrotactile tasks, respectively, while panels C and D show the lapse rates for the color and vibrotactile tasks. Download Figure 5-1, ZIP file. [file eneuro-12-ENEURO.0121-25.2025-s002.zip › PsychometricFits_Vibrotactile/Psychometric_8785.png]

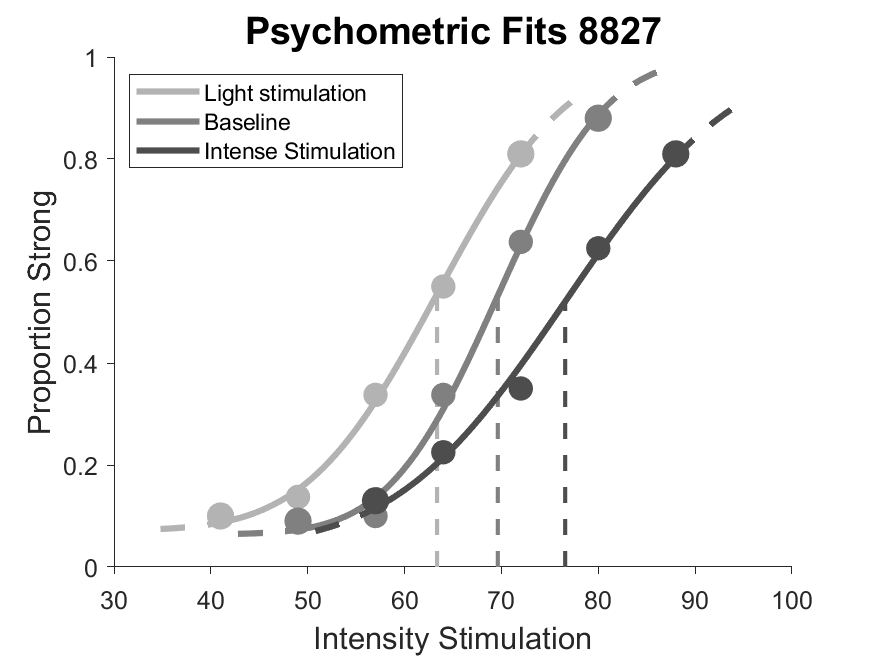

Supplement: Figure 5-1 — Guess and lapse rates for the color and vibrotactile tasks. Panels A and B show the guess rates for the color and vibrotactile tasks, respectively, while panels C and D show the lapse rates for the color and vibrotactile tasks. Download Figure 5-1, ZIP file. [file eneuro-12-ENEURO.0121-25.2025-s002.zip › PsychometricFits_Vibrotactile/Psychometric_8827.png]

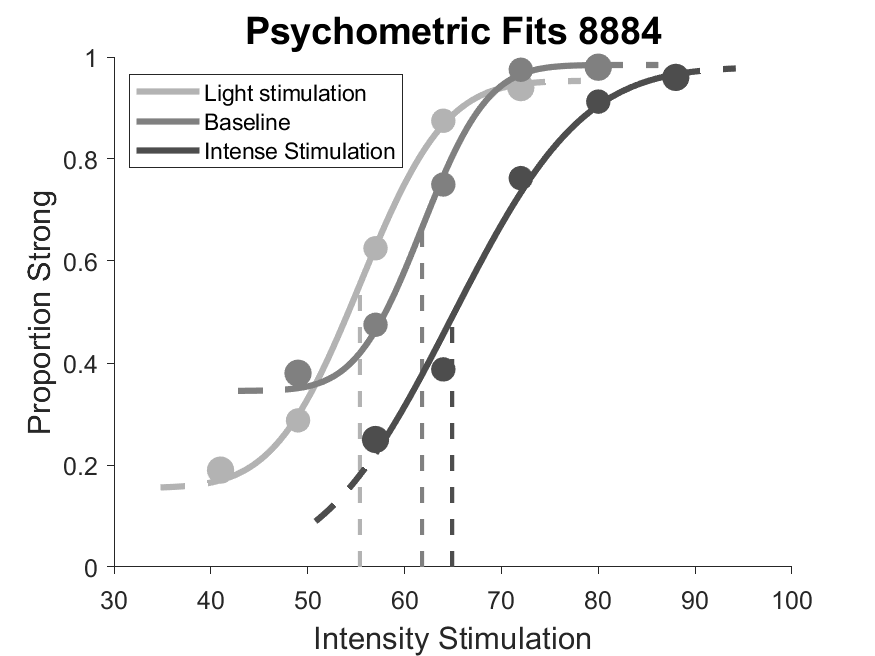

Supplement: Figure 5-1 — Guess and lapse rates for the color and vibrotactile tasks. Panels A and B show the guess rates for the color and vibrotactile tasks, respectively, while panels C and D show the lapse rates for the color and vibrotactile tasks. Download Figure 5-1, ZIP file. [file eneuro-12-ENEURO.0121-25.2025-s002.zip › PsychometricFits_Vibrotactile/Psychometric_8884.png]
